# Electromagnetic all-in-one radiation-scattering reconfigurable intelligent metasurface

**DOI:** 10.1093/nsr/nwaf470

**Published:** 2025-11-03

**Authors:** Yajie Mu, Jiaqi Han, Hao Xue, Qiang Feng, Lingyun Niu, Haixia Liu, Long Li

**Affiliations:** Key Laboratory of High-Speed Circuit Design and EMC of Ministry of Education, School of Electronic Engineering, Xidian University, Xi’an 710071, China; Key Laboratory of High-Speed Circuit Design and EMC of Ministry of Education, School of Electronic Engineering, Xidian University, Xi’an 710071, China; Key Laboratory of High-Speed Circuit Design and EMC of Ministry of Education, School of Electronic Engineering, Xidian University, Xi’an 710071, China; Key Laboratory of High-Speed Circuit Design and EMC of Ministry of Education, School of Electronic Engineering, Xidian University, Xi’an 710071, China; Key Laboratory of High-Speed Circuit Design and EMC of Ministry of Education, School of Electronic Engineering, Xidian University, Xi’an 710071, China; Key Laboratory of High-Speed Circuit Design and EMC of Ministry of Education, School of Electronic Engineering, Xidian University, Xi’an 710071, China; Key Laboratory of High-Speed Circuit Design and EMC of Ministry of Education, School of Electronic Engineering, Xidian University, Xi’an 710071, China

**Keywords:** all-in-one, multifunctional and multi-scenario applications, radiation-scattering design, reconfigurable intelligent metasurface

## Abstract

Reconfigurable intelligent surfaces (RISs) bring new opportunities for various fields. Currently, RISs can only be used in specific scenarios, such as wireless communication in scattering mode. It is difficult to integrate radiation and scattering modes in a single RIS for multi-scenario applications. To achieve integrated hardware design for multifunctional and multi-scenario applications while reducing hardware costs and size, we propose an electromagnetic all-in-one RIS. A simple and flexible radiation-scattering design framework is constructed, which can realize a radiation-scattering meta-atom design with on-demand polarization and phase—that is, an all-in-one radiation-scattering meta-atom. The meta-atom consists of a radiating patch and a 3-dB coupler. The radiating patch determines the polarization characteristics. The diodes are loaded on the 3-dB coupler to realize the radiation-scattering mode switching and the corresponding mode phase control. By loading different capacitors on the radiating patch, this design also integrates initial radiation and scattering phases into a single structure for the first time. The meta-atoms with specific polarization exhibit amplitude control, phase control and customizable initial phase properties. Specifically, loading PIN diodes or varactor diodes on the 3-dB coupler enables 1-bit or continuous phase regulation, respectively. Under stringent hardware constraints and limited physical space, the radiation-scattering RIS provides multifunctional capabilities within a single platform. The RIS can achieve cost-effective phased arrays in radiation mode. Non-line-of-sight communication is achieved in scattering mode. A $12\times 12$ radiation-scattering RIS was fabricated to demonstrate its performance. This framework bridges a critical gap in electromagnetic radiation-scattering systems by enabling arbitrary polarization and phase modulation, offering a promising technical solution for 6G wireless communication.

## Teaser

An electromagnetic all-in-one RIS integrates radiation and scattering modes through a single and flexible design framework, which enables a radiation-scattering design with on-demand polarization and phase for versatile 6G applications.

## INTRODUCTION

Sixth-generation (6G) wireless communication technology is expected to enable cutting-edge applications such as the Internet of Everything. However, existing communication systems, constrained by limited resources, struggle to meet user demands for high data rates, low latency and seamless connectivity. Moreover, ongoing research and development of millimeter-wave technology for prospective 6G applications may introduce challenges such as increased path loss and coverage of blind spots [1]. Against this backdrop, reconfigurable intelligent surfaces (RISs), characterized by low cost, low loss, programmability and ease of deployment, emerge as a powerful solution for wireless communication networks, significantly enhancing network coverage [2–4].

The RIS is a two-dimensional artificial metasurface composed of periodic or non-periodic subwavelength electromagnetic resonant units [5]. Unlike static metasurfaces, RIS units can dynamically alter the amplitude and phase response of reflected/transmitted waves by incorporating components such as PIN and varactor diodes, enabling dynamic beamforming. RIS primarily consists of reflection, transmission and reflection–transmission integrated reconfigurable metasurfaces. The phase-control precision of RIS is mainly 1-bit and 2-bit. The research trend for RISs focuses on improving phase quantization precision to achieve high-performance control of linearly or circularly polarized electromagnetic waves. As future wireless communication scenarios diversify, RISs also evolve towards multifunctionality. They integrate amplitude/polarization modulation with phase modulation [6,7]. Moreover, sensors on metasurfaces enable adaptive information transmission and processing [8,9].

Compared to the high-cost phase shifters and Transmitter-Receiver modules in phased arrays, an RIS offers an economical alternative. As a low-cost phased array, an RIS can achieve target detection and tracking [10,11], and localization [12,13], among others. An RIS also serves as a powerful supplement to wireless communication systems, effectively enhancing the coverage, spectral efficiency and energy efficiency of existing wireless networks. In non-line-of-sight (NLOS) scenarios, signals may attenuate or distort due to the nature of NLOS propagation. An RIS can intelligently alter the signal propagation path to reach signal dead zones [14–17]. Therefore, communication can be maintained even when the line of sight (LOS) is blocked. In LOS scenarios, an RIS can achieve beamforming by adjusting the phase and amplitude of the reflected signals [18,19]. By focusing the signal energy in a specific direction, an RIS improves transmission efficiency and coverage accuracy in LOS scenarios. An RIS offers greater optimization potential and possibilities for future wireless communication networks [20]. Moreover, an RIS is also employed in wireless energy transmission and harvesting systems [21–24]. An RIS dynamically adjusts the direction of energy transfer and harvesting to ensure efficient delivery to mobile terminals.

However, 6G wireless communication technology, which aims to connect everything intelligently, requires an RIS to possess both communication and sensing capabilities [25–27]. This involves integrating radar and communication functions. This necessitates integrating sensing and communication functions on a single hardware platform to optimize resource allocation and reduce hardware costs. Currently, most existing RISs are reflection [28–32], transmission [33,34] or integrated reflection–transmission types [35,36]. These are primarily designed to enhance the quality and coverage of wireless communication networks. The hardware limitations of existing RISs are hindering the development of wireless communication technologies. There is an urgent need for a new hardware paradigm to break through these limitations. Most RISs focus on controlling scattered electromagnetic waves, with few addressing radiated waves. Programmable phased arrays achieve control of radiated electromagnetic waves by modulating PIN diodes loaded on antenna elements [37–44]. Recent studies have integrated programmable phased arrays with RISs to control both scattered and radiated electromagnetic waves [45–49]. This integrated design can serve as a new hardware paradigm for 6G wireless communication technology, enabling unified communication and sensing. However, existing integrated radiation-scattering RISs have complex structures and large phase quantization errors. Enhancing the phase quantization precision of integrated radiation-scattering RISs is extremely challenging. This limits their performance in practical applications. Specifically, both 1-bit scattering and 1-bit radiation beam control result in grating lobes, reducing efficiency. Ning *et al.* [45] achieved 1-bit phase control of radiation and scattering using three PIN diodes. Tian *et al.* [46] achieved 1-bit phase control of radiation and scattering using two PIN diodes and two varactor diodes. Clearly, an integrated radiation-scattering RIS requires numerous RF components, resulting in complex structures and high losses. Although Mu *et al.* [47] achieved 2-bit phase control of radiation using four PIN diodes, the scattering phase remained 1-bit. More importantly, current radiation-scattering RIS design frameworks are complex and can only achieve single linear polarization. They are incapable of arbitrary polarization and phase design.

To achieve the lowest hardware cost and optimal benefit, we propose an electromagnetic all-in-one radiation-scattering RIS. A simple and flexible radiation-scattering meta-atom design framework is constructed, which can realize radiation-scattering meta-atom design with arbitrary polarization and phase. The radiation-scattering RIS meta-atom consists of a radiating patch and a 3-dB coupler. The patch and 3-dB coupler are responsible for polarization and phase/amplitude characteristics, respectively. The specific patch is linearly polarized, and two PIN diodes are loaded on the 3-dB coupler. One-bit scattering and 1-bit radiation phase control can be achieved. By loading capacitors of different values onto the patch, we designed four types of radiating units with distinct initial phases. For the first time, we integrated four initial radiation and scattering phases into a single design. Arrays composed of these four types of radiation-scattering meta-atoms can suppress grating lobes caused by quantization errors in 1-bit scattering and 1-bit radiation, thereby improving efficiency. Moreover, these four initial phases can achieve high-performance beam scanning without the need for any multi-objective optimization algorithms to optimize their positions. The 1-bit radiation design enables cost-effective phased arrays, while the 1-bit scattering design supports NLOS communication. Furthermore, the framework enables continuous phase modulation of meta-atoms when two varactor diodes and one PIN diode are loaded on a 3-dB coupler. Additionally, it reveals the radiation-scattering characteristics of meta-atoms under a circularly polarized patch. This demonstrates the flexibility and multifunctionality of our antenna and circuit decoupling framework. A $12\times 12$ linearly polarized radiation-scattering RIS with 1-bit phase control was fabricated to demonstrate its phased array, and NLOS blind-spot coverage capabilities. The proposed RIS integrates radiation and scattering into a single hardware platform, enabling multiple functions and providing more options for next-generation wireless communications.

## RESULTS

### Concept of a radiation-scattering reconfigurable intelligent metasurface

**Figure 1.**
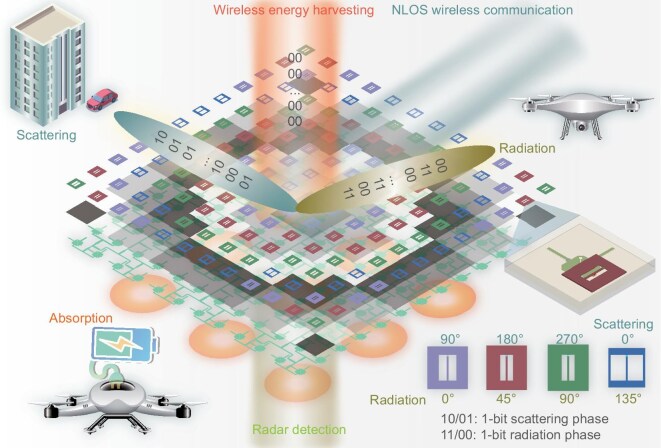
Schematic illustration of an all-in-one radiation-scattering RIS. When the PIN diodes are in states 11 and 00, 1-bit radiation phase control is achieved. When the PIN diodes are in states 10 and 01, 1-bit scattering phase control is achieved.

We propose an electromagnetic all-in-one radiation-scattering RIS, as shown in Fig. 1. Using linearly polarized 1-bit radiation-scattering meta-atoms as an example, we explore their functionalities and potential applications. The meta-atom consists of a U-slot patch and a 3-dB coupler connected via metalized vias. The PIN diode is loaded on both the through and coupled branches of the 3-dB coupler. The off state of the PIN diode is defined as 0, and the on state as 1. When the PIN diodes are in states 11 and 00, 1-bit radiation phase control is achieved. When the PIN diodes are in states 10 and 01, 1-bit scattering phase control is achieved. However, both 1-bit radiation and scattering beam control result in grating lobes due to quantization errors. This severely impacts the performance of the 1-bit RIS. Therefore, we load capacitors on the radiating patch, with different capacitors corresponding to different initial phases. Ultimately, we designed four types of U-slot patches loaded with different capacitors. This achieves initial scattering phases of 0$^{\circ }$, 90$^{\circ }$, 180$^{\circ }$ and 270$^{\circ }$, and initial radiation phases of 0$^{\circ }$, 45$^{\circ }$, 90$^{\circ }$ and 135$^{\circ }$. This is because the scattering path is twice that of the radiation path; hence, the initial phase is twice the radiation phase. In this paper, we adopt the method of calculating phase delays introduced by near-field horn excitation in reflectarrays to select the initial phase distribution. Then, the initial phase distribution is mapped onto four types of meta-atoms with different initial phases to form a $12\times 12$ radiation-scattering RIS. It is precisely because four initial phases are introduced in both radiation and scattering modes that grating lobes in 1-bit radiation and scattering RISs are suppressed. When the 1-bit scattering RIS is used for NLOS communication, the initial phases are quantized to 0$^{\circ }$, 90$^{\circ }$, 180$^{\circ }$ and 270$^{\circ }$. When the 1-bit radiation RIS is used as a phased array, the initial phases are quantized to 0$^{\circ }$, 45$^{\circ }$, 90$^{\circ }$ and 135$^{\circ }$. Moreover, when the PIN diodes are in the 00 state, the radiation-scattering RIS can perform wireless energy harvesting (WEH). The harvested energy, after rectification, can be used to charge other electronic devices or supply power to the RIS itself. Thus, the proposed RIS integrates radiation and scattering into a single hardware platform, enabling multiple functions and providing more options for next-generation wireless communications.

### All-in-one radiation-scattering meta-atom design

**Figure 2.**
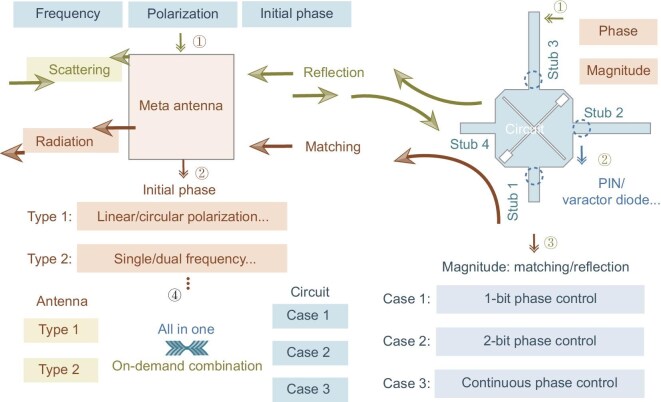
All-in-one radiation-scattering meta-atom design framework.

The radiation-scattering meta-atom design employs a principle where the antenna terminal receives signals and the circuit terminal regulates functionality. This approach simplifies the design complexity of radiation-scattering meta-atoms. Within this framework, diverse radiation-scattering meta-atom designs can be implemented with enhanced simplicity and flexibility, as shown in Fig. 2. The design framework comprises three main components. The first component is the front-end antenna design. The primary function of the antenna is to achieve impedance matching with the back-end circuit. Consequently, there are no inherent restrictions on the antenna type, allowing for selection from a wide variety of designs. Antennas can be designed based on characteristics such as operating frequency, polarization and phase response. Through a combination of these characteristics, the framework supports the realization of single-band linearly or circularly polarized antennas, as well as dual-band linearly or circularly polarized antennas. The second component is the back-end circuit design. The core function of this circuit is to regulate magnitude and phase. A 3-dB coupler serves as the core circuit element. Amplitude control is achieved by switching the state of stub 4 (connected to the antenna) between matched and reflection conditions, thereby toggling the meta-atom between radiation and scattering modes. Phase control is implemented by modulating the electrical lengths of stub 2 and stub 3. Thus, by loading stub 2 and stub 3 with either PIN or varactor diodes, the phase of the signal output at stub 4 from the 3-dB coupler can be controlled. When the states of the two PIN diodes are set to 11 or 00, stub 4 is matched, and the output signals exhibit a 180$^{\circ }$ phase difference. When the PIN diode states are 01 or 10, stub 4 is reflective, and the reflected signals exhibit a 180$^{\circ }$ phase difference. Therefore, incorporating PIN diodes on stub 2 and stub 3 enables simultaneous control over both amplitude and phase. Although only two PIN diodes are required, this configuration is limited to 1-bit phase control for both radiation and scattering states. Replacing the PIN diodes with varactor diodes on stub 2 and stub 3 enables continuous phase tuning. In this configuration, the two varactor diodes are biased identically. Consequently, this results in stub 4 remaining matched, precluding a reflection state. To switch stub 4 between matched and reflection states, an additional PIN diode is required on stub 1. A 2-bit phase-control design operates on a similar principle to the continuous-phase-control scheme. The third component involves the integration of the selected front-end antenna with the back-end circuit. By selecting the desired characteristics from antenna and circuit libraries, an on-demand design process is realized. Therefore, this decoupled front-end/back-end framework achieves an all-in-one radiation-scattering meta-atom design in a highly streamlined manner. Without modifying back-end circuits, radiation-scattering meta-atoms supporting linear, circular or dual polarization can be realized solely by designing antenna terminals with corresponding polarization properties. By modifying the diode-loading method and quantity on the 3-dB coupler without altering the antenna, continuous phase modulation of radiation-scattering meta-atoms is achieved. The decoupled antenna-circuit framework liberates antenna design from phase control or mode-switching constraints, thereby improving flexibility. Thus, antennas with diverse initial phases can be readily designed. As electromagnetic waves for both modes are received by the antenna and transmitted to the circuit, dual initial phases (i.e. distinct phases for radiation/scattering modes) become feasible. Here, we demonstrate this framework using a linearly polarized antenna to design radiation-scattering meta-atoms with tunable circuits.

**Figure 3.**
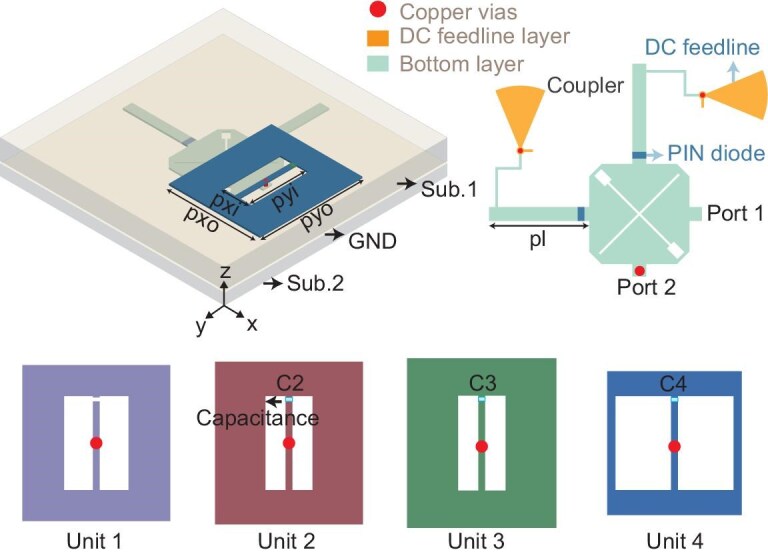
Geometry of the meta-atom. Four meta-atoms: Unit 1, 2, 3 and 4.

The proposed linearly polarized radiation-scattering meta-atom is shown in Fig. 3. The meta-atom consists of two dielectric substrates and four metallic layers. The two dielectric substrates are bonded with a 0.2-mm-thick layer of Rogers 4350F in between. The thicknesses of substrates 1 and 2 are 1.524 and 0.508 mm, respectively, with a dielectric constant of 3.55 and a loss tangent of 0.005. The four metallic layers, from top to bottom, are the U-slot patch, the metallic ground, the DC feed layer and the 3-dB coupler. Metalized vias are used to connect the U-slot patch to the 3-dB coupler while isolating them from the ground plane. The U-slot patch is a microstrip antenna capable of receiving and transmitting RF signals. A PIN diode is loaded on both the through and coupled branches of the 3-dB coupler. The PIN diodes are SMP1340. A quarter-wavelength high-impedance line and a fan-shaped stub are extended from the sides of the through and coupled branches, serving as the DC feedlines for the two PIN diodes. The use of quarter-wavelength high-impedance lines and fan-shaped stubs is to minimize the impact of the DC feedlines on RF signals. The DC feedlines are connected to the upper layer of substrate 2 via metalized vias, serving as the DC feed network layer for the array. Compared to conventional 3-dB couplers, the through and coupled branches of the proposed meta-atom are longer. This design ensures that the phase difference between the signal transmitted from port 1 to port 2 and then radiated by the U-slot patch is approximately 180$^{\circ }$ in 00 and 11 states, compared to 90$^{\circ }$ in conventional cases. When the PIN diodes are in states 01 and 10, the U-slot patch receives the incident wave and transmits it to port 1. Port 1 reflects the received electromagnetic wave. Since the reflection phase at port 1 differs by approximately 180$^{\circ }$ in states 01 and 10 of the 3-dB coupler, 1-bit scattering phase control is achieved. Thus, by controlling the states of the two PIN diodes on the 3-dB coupler, the meta-atom achieves 1-bit phase control of both radiated and scattered electromagnetic waves. Additionally, four types of U-slot antennas were designed: Unit 1, Unit 2, Unit 3 and Unit 4. All four antennas operate within the same frequency band. However, they have different sizes and are loaded with different capacitance values to achieve distinct initial phases. The capacitance values loaded on Unit 1, Unit 2, Unit 3 and Unit 4 are 0, 0.1, 0.2 and 0.5 pF, respectively. The four U-slot antennas are terminated with a 3-dB coupler, thus realizing a 1-bit radiation-scattering meta-atom design with four initial phases. Detailed parameter designs are provided in Section S1.

**Figure 4.**
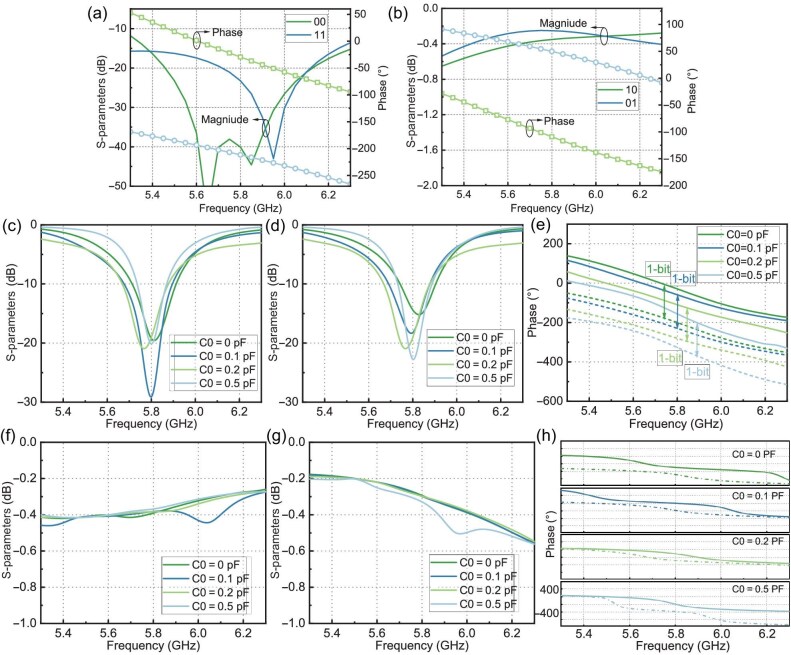
Simulation results of the meta-atom. (a, b) The *S*-parameters of the 3-dB coupler loaded with PIN diodes in (a) matched and (b) reflective modes. (c, d) Reflection amplitude of the meta-atom in the radiation mode for (c) 00 and (d) 11 states. (e) Radiation phase of the meta-atom in the radiation mode. (f, g) Reflection amplitude of the meta-atom in the scattering mode for (f) 01 and (g) 10 states. (h) Phase of the meta-atom in the scattering mode.

The amplitude and phase of the *S*-parameters for port 1 of the 3-dB coupler in matched and reflection states are shown in panels (a) and (b) of Fig. 4. The *S*-parameters for port 2 in matched and reflection states are provided in Fig. S1a within the online supplementary material. When the PIN diodes are in states 00 and 11, ports 1 and 2 are matched, as shown in Fig. 4a. In these states, the *S*-parameters of ports 1 and 2 are identical. It can be seen that the reflection coefficients at 5.3–6.3 GHz are below $-10$ dB for both states 00 and 11. At 5.7–5.9 GHz, the transmission phase difference between states 00 and 11 is $180^{\circ }\pm 20^{\circ }$. The amplitude and phase of the *S*-parameters for port 1 of the 3-dB coupler in full reflection are shown in Fig. 4b. The *S*-parameters for port 2 in full reflection are provided in Fig. S1b within the online supplementary material. It can be seen that the reflection coefficients at 5.3–6.3 GHz are above $-0.8$ dB for states 10 and 01. The reflection phase difference between these two states at 5.7–5.9 GHz satisfies $180^{\circ }\pm 20^{\circ }$. The matching of the U-slot antennas without the 3-dB coupler is shown in Fig. S2. It can be seen that the port reflection coefficients of the four U-slot antennas (Unit 1, Unit 2, Unit 3 and Unit 4) are below $-10$ dB at 5.7–5.9 GHz, indicating good matching. Moreover, the radiation phases of these four antennas differ by approximately $45^{\circ }$.

By connecting the ports of the four U-slot antennas (Unit 1, Unit 2, Unit 3 and Unit 4) to port 2 of the 3-dB coupler, we constructed the 1-bit radiation-scattering meta-atom. Detailed design principles are provided in Section S2. When the PIN diodes are in states 00 and 11, the meta-atom operates in the radiation mode. The magnitude and phase of the *S*-parameters for these four meta-atoms are shown in Fig. 4c–e. It can be seen that the matching bandwidth for these four meta-atoms in states 00 and 11 is 5.7–5.9 GHz. Notably, the radiation phase difference between states 00 and 11 for these four meta-atoms within 5.7–5.9 GHz satisfies $180^{\circ }\pm 20^{\circ }$. Moreover, the phase difference between Unit 1, Unit 2, Unit 3 and Unit 4 is approximately $45^{\circ }$, achieving an initial phase distribution of 0$^{\circ }$, 45$^{\circ }$, 90$^{\circ }$ and 135$^{\circ }$.

When the PIN diodes are in states 01 and 10, the meta-atom operates in the scattering mode. In states 10 and 01, the scattering phase differs by 1-bit, endowing the meta-atom with 1-bit phase-control capability in the scattering mode. In the scattering mode, the amplitude and phase of the *S*-parameters for the four meta-atoms (Unit 1, Unit 2, Unit 3 and Unit 4) are shown in Fig. 4f–h. It can be seen that the reflection coefficients for these four meta-atoms in states 01 and 10 are greater than $-0.64$ dB within 5.7–5.9 GHz, indicating total reflection. Notably, the reflection phase difference between states 01 and 10 for these four meta-atoms within 5.7–5.9 GHz satisfies $180^{\circ }\pm 20^{\circ }$. Moreover, the phase difference between Unit 1, Unit 2, Unit 3 and Unit 4 is approximately $90^{\circ }$, achieving an initial phase distribution of 0$^{\circ }$, 90$^{\circ }$, 180$^{\circ }$ and 270$^{\circ }$. The initial phases of the four units in both radiation and scattering modes are provided in Tables S3 and S4. The impact of the PIN diode’s on-state resistance on the radiation and scattering performance of meta-atoms is elaborated on in Section S3. The designs for the 1-bit circularly polarized and the continuously tunable linearly polarized meta-atoms are presented in Sections S4 and S5.

### Radiation-scattering reconfigurable intelligent metasurface design

The far-field pattern of the $M\times N$ array can be written as


(1) \begin{eqnarray*}
E(\theta , \varphi )&=& \sum _{n=1}^{N} \sum _{m=1}^{M} f_{m n}(\theta , \varphi ) \\
&& \times e^{-j(\varphi (m, n)+k m d \sin \theta \cos \varphi +k n d \sin \theta \sin \varphi )}, \\
\end{eqnarray*}

where $\theta$ and $\varphi$ are the elevation and azimuth angles of the main beam, respectively, $f(\theta , \varphi )$ is the element factor, $\varphi (m, n)$ is the phase of the $(m, n)$th element, *d* is the element periodicity and $k_0$ is the wave number. For an array with deflection angle $(\theta , \varphi )$, the required continuous phase compensation for the $(m, n)$th element is


(2) \begin{eqnarray*}
\varphi (m, n) = -k m d \sin \theta \cos \phi - k n d \sin \theta \sin \phi.\\
\end{eqnarray*}

For an array with a given initial phase $\varphi _0(m, n)$, the phase compensation required for the $(m, n)$th element with the beam pointing at $(\theta , \varphi )$ is


(3) \begin{eqnarray*}
\varphi (m, n)&=& -k m d \sin \theta \cos \phi \\
&& - k n d \sin \theta \sin \phi -\varphi _0(m, n).
\end{eqnarray*}

For a 1-bit meta-atom, only the 0$^{\circ }$ and 180$^{\circ }$ options are available instead of a continuous phase. The continuous phase $\varphi (m, n)$ is discretized as


(4) \begin{eqnarray*}
\varphi (m,n)= \left\lbrace \begin{array}{@{}l@{\quad }l@{}}0, &\quad 0^\circ \le \varphi < 180^\circ ,\\
180^\circ , &\quad 180^\circ \le \varphi < 360^\circ . \end{array}\right.
\end{eqnarray*}

In the radiation mode, the emitted electromagnetic wave can be approximated as a plane wave. Consequently, the initial phases of the $M\times N$ meta-atoms in the array are identical and defined as a relative value of 0$^{\circ }$. This results in the characteristic of dual-beam scanning due to 1-bit phase quantization. In the traditional scattering mode, a horn located in the near-field region of the array is generally used as the excitation source. This causes the phase distribution of the electromagnetic wave upon reaching the array surface to be non-uniform, resembling a spherical wave with a certain initial phase $\varphi _f(m, n)$. The phase delay caused by the feed source can be expressed as


(5) \begin{eqnarray*}
\varphi _f = {k}\sqrt{(x - x_0)^2 + (y - y_0)^2 + (z - z_0)^2},\\
\end{eqnarray*}

where $(x, y, z)$ represents the position of each element and $(x_0, y_0, z_0)$ is the position of the feed source with $x_0 = 0, y_0 = 0, z_0 = F$. The focal diameter ratio (FDR) is defined as $F/D$, where *D* is the size of the array. Therefore, when the horn excites the array in the near-field region, the corresponding phase compensation for the meta-atoms during beam scanning is


(6) \begin{eqnarray*}
\varphi (m, n) &=& -k m d \sin \theta \cos \phi \\
&& - \,k n d \sin \theta \sin \phi -\varphi _f(m, n).
\end{eqnarray*}

Subsequently, beam scanning is achieved through 1-bit phase quantization. However, for NLOS blind-spot coverage, the horn is positioned in the far-field region. This causes the electromagnetic wave to approximate a plane wave, with an initial phase of 0$^{\circ }$. One-bit phase quantization also results in the characteristic of dual-beam scanning. To suppress the dual-beam issue in both radiation and scattering modes, the meta-atoms must possess initial phases in both modes. Therefore, we propose meta-atoms with four distinct initial phases. On this paper, we adopt the calculation of the phase delay introduced by the near-field horn in the reflectarray as the method for selecting the initial phase distribution, namely Equation (5). The only difference is that in traditional designs, the initial phase is introduced by the spherical wave excited by the near-field horn. We achieve the initial phases through four distinct meta-atoms, without the need for near-field horn excitation. This significantly reduces the profile height. Therefore, the phase compensation for each unit in the array still employs Equation (6). The initial phase design principles are elaborated on in Section S6.

The designed meta-atoms are assembled into a $12\times 12$ RIS array. The unit-cell period is 25 mm. The two PIN diodes on the 3-dB coupler are controlled by two DC feedlines. The DC feedlines are located on the sides of the through and coupled branches of the 3-dB coupler. High-impedance fan-shaped stubs are used to suppress the impact of the DC feedlines on the RF signals. The input of the 3-dB coupler is connected to the DC ground via a fan-shaped stub. A total of 288 DC control signals are required. The topology of the $12\times 12$ RIS array is provided in Fig. S8.

### Cost-effective phased array

**Figure 5.**
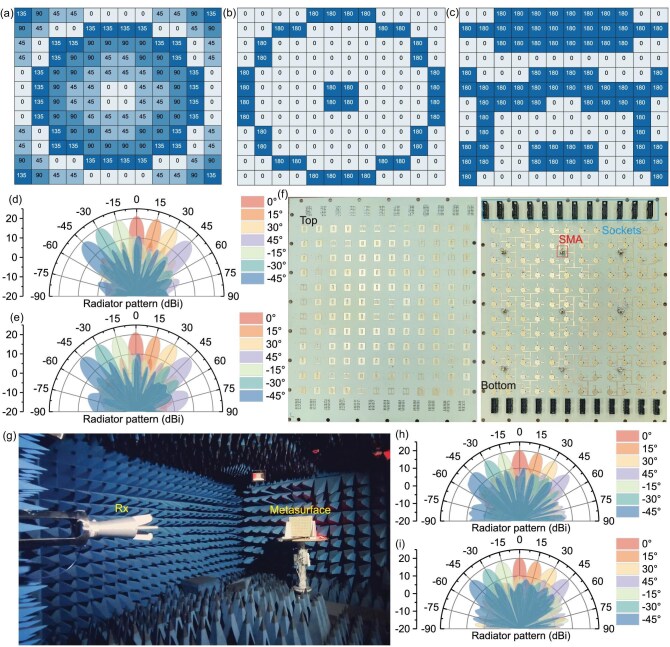
Experimental results of the radiation-scattering RIS. (a) Initial phases in the radiation mode. (b, c) Coding matrices for radiation beams scanned to (b) (0$^{\circ }$, 0$^{\circ }$) and (c) (0$^{\circ }$, 30$^{\circ }$). (d, e) Beam-scanning results in the radiation mode. (f) Photograph of the fabricated array. (g) Test setup for the radiation mode. (h, i) Radiation mode test results at 5.8 GHz: (g) E plane and (i) H plane.

The initial phase selection is calculated based on Equation (5). The FDR is set to 0.6, and the array size is $12\times 12$. The initial phase required for each unit is obtained through Equation (5), as shown in Fig. S9a. The resulting phases have a large number of distinct values. Designing metasurface units with such a large number of initial phases would be not only complex but also difficult to implement. Therefore, we quantize the initial phases to 2-bit levels. The quantized initial phases are shown in Fig. S9b. After 2-bit quantization, only four initial phases remain: 0$^{\circ }$, 90$^{\circ }$, 180$^{\circ }$ and 270$^{\circ }$. This greatly simplifies the design complexity of the initial phases. For the radiation mode, the four initial phases are 0$^{\circ }$, 45$^{\circ }$, 90$^{\circ }$ and 135$^{\circ }$. For the scattering mode, the four initial phases are 0$^{\circ }$, 90$^{\circ }$, 180$^{\circ }$ and 270$^{\circ }$. Specifically, the initial phases for radiation and scattering for Unit1, Unit2, Unit3 and Unit4 are [0$^{\circ }$, 90$^{\circ }$], [45$^{\circ }$, 180$^{\circ }$], [90$^{\circ }$, 270$^{\circ }$] and [135$^{\circ }$, 0$^{\circ }$], respectively. Since the scattering initial phases satisfy 2-bit quantization, the obtained initial phases are mapped onto the four scattering meta-atoms to form the array topology, as shown in Fig. S9c. Once the array topology is determined, the initial phase distribution for the radiation mode is also established, as shown in Fig. 5a.

The corresponding phases of the meta-atoms at different scanning angles in radiation mode are then obtained through Equation (6). Figure 5b, c show the coding matrices when the beam is deflected to (0$^{\circ }$, 0$^{\circ }$) and (0$^{\circ }$, 30$^{\circ }$), respectively. Figure 5d, e present the beam-scanning results of the array in the E plane and H plane. It can be seen that within the $\pm 45^{\circ }$ beam-scanning range, the gain ranges from 21.33 to 18.80 dBi. Notably, in the radiation mode, the 1-bit RIS no longer exhibits dual-beam characteristics during beam scanning. Moreover, within the $\pm 45^{\circ }$ beam-scanning range, the side-lobe level is greater than or equal to 10 dB. Beam-scanning results for other frequencies are provided in Fig. S10. To highlight the advantages of the initial phase design, the beam characteristics of the RIS without initial phase are provided in Fig. S12. The $12\times 12$ RIS array was fabricated using PCB technology. The fabricated model is shown in Fig. 5f. The beam-scanning characteristics in the radiation mode were tested in the microwave anechoic chamber shown in Fig. 5g. A 1-to-9 power divider was used to combine the signals from the nine ports of the array. The characteristics of the 1-to-9 power divider are provided in Fig. S13. The tested E-plane and H-plane beam-scanning results at 5.8 GHz are shown in panels (h) and (i) of Fig. 5, respectively. It can be seen that the tested and simulated characteristics are consistent. Single-beam scanning can be achieved within the $\pm 45^{\circ }$ beam-scanning range, with a side-lobe level greater than or equal to 10 dB. The gain fluctuation range is 20.35–18.1 dBi. The test results for other frequencies are provided in Fig. S11. A detailed analysis of the impact of a 1-to-9 power-divider feeding mechanism on RIS radiation performance is provided in Section S11. The phase and amplitude consistency errors of the 1-to-9 power divider exert a negligible impact on RIS performance.

**Figure 6.**
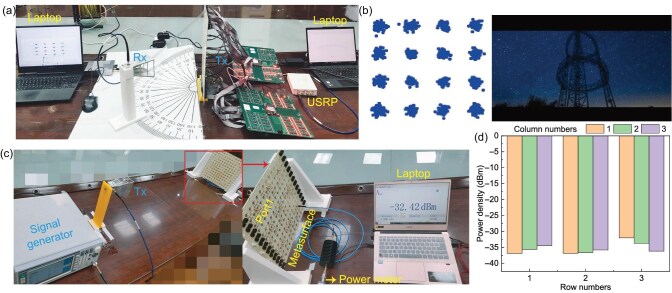
Communication and the WEH experiment. (a) Setup for the communication scenario. (b) Sixteen-QAM constellation diagram and video transmission results. (c) Test setup for WEH. (d) Power density distribution collected at the ports.

The proposed RIS was applied in communication experiments to verify its performance. Figure 6a shows the communication experiment setup, where 16-QAM modulation was used for video transmission. One USRP was used to connect to the input port of the RIS, while the other USRP was connected to the receiving horn antenna. Figure 6b presents the constellation diagram and video transmission performance when the RIS beam was directed at 30$^{\circ }$ and the receiving horn was located at 30$^{\circ }$. The constellation diagram maintains a clear and regular shape. Therefore, the proposed RIS exhibits excellent characteristics, with low cost and design complexity. It can serve as a cost-effective phased array for future 6G wireless communication.

Furthermore, RISs in radiation mode can also serve as receivers. They are applied for sensing or WEH. Subsequently, we analyze WEH characteristics. The reflection coefficients of the nine ports of the proposed $12\times 12$ RIS are provided in Fig. S16. It can be seen that, within 5.7–5.9 GHz, the reflection coefficients are below $-10$ dB, indicating good matching. Therefore, the experimental setup shown in Fig. 6c was used to test the energy received by the nine ports of the $12\times 12$ RIS. A signal generator was connected to the transmitting horn to emit a 5.8-GHz signal with an input power of 0 dBm. The RIS was placed 1.5 m away from the transmitting horn. The received power at each port was measured using a power probe. The received power values for the nine ports are shown in Fig. 6d. The maximum received power at the ports was $-32.42$ dBm, while the minimum was $-38.6$ dBm. Therefore, the proposed RIS is capable of WEH without the need for active PIN diodes. By connecting the energy collected from the nine ports to a rectifier circuit, the RF energy can be converted into DC energy.

### NLOS wireless communication

**Figure 7.**
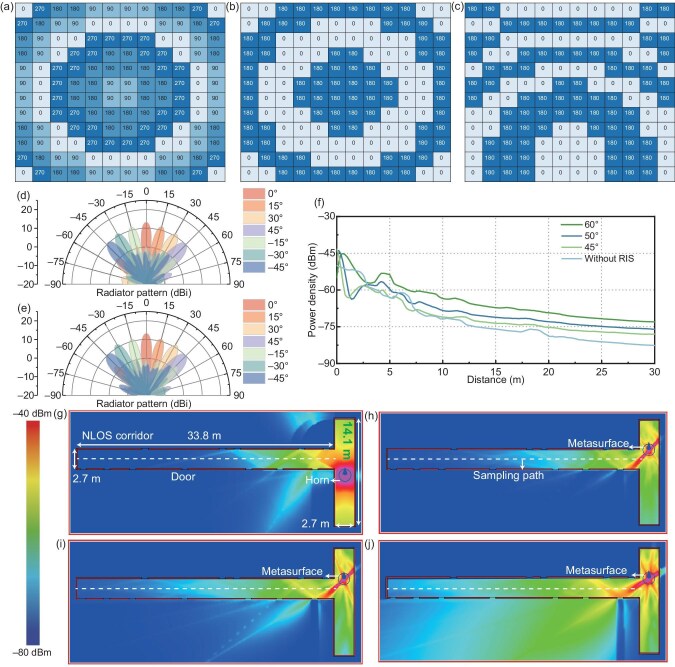
Experimental results of the RIS in scattering mode. (a) Initial phases. (b, c) Coding matrices for scattering beams scanned to (b) $(0^\circ , 0^\circ )$ and (c) $(0^\circ , 30^\circ )$. (d, e) Simulated beam-scanning results in the (d) E plane, and (e) H plane. (f) Blind-spot coverage characteristics in the L-shaped corridor. (g–j) Power density distribution in the L-shaped corridor: (g) without an RIS, (h) RIS beam at 45$^{\circ }$, (i) RIS beam at $50^\circ$ and (j) RIS beam at $60^\circ$.

The proposed RIS suppresses grating lobes by introducing initial phases. The initial phases of the proposed RIS in scattering mode are shown in Fig. 7a. The method for designing initial phases is described above. With these initial phases, the required phases for different beam directions are obtained through Equation (6). Figure 7b, c show the coding matrices for scattering beams at (0$^{\circ }$, 0$^{\circ }$) and (0$^{\circ }$, 30$^{\circ }$), respectively. Figure 7d, e present the E-plane and H-plane beam-scanning results of the proposed RIS at 5.8 GHz under plane-wave incidence. Notably, grating-lobe-free single-beam scanning is achieved within the $\pm 45^{\circ }$ beam-scanning range. The maximum gain is 13.33 dBi, with a gain fluctuation of 3 dB. The side-lobe level is greater than or equal to 10 dB. Beam-scanning results for other frequencies are provided in Fig. S17. To highlight the advantages of the initial phase design, the beam characteristics of RISs without initial phase are provided in Fig. S19. Therefore, the introduction of initial phases enhances the beam-scanning performance of 1-bit scattering RISs.

We investigate the NLOS wireless communication scenario in an L-shaped corridor within a building. Figure 7f shows the power density distribution in the L-shaped corridor of the teaching building with and without the installation of an RIS. The L-shaped corridor and its power distribution are shown in Fig. 7g–j. The height of the L-shaped corridor is 3.8 m, and the width is 2.7 m. The length of the corridor on the NLOS side is 33.8 m, and on the LOS side is 14.1 m. The walls are made of 20-cm-thick concrete. There are 12 doors in the scene, all made of metal. The height of the doors is 2.95 m, and the width is 0.97 m. First, a horn antenna is used as the transmitter to simulate the scenario of an active base station. The power intensity distribution of the aforementioned L-shaped scenario is analyzed. The height of the transmitting horn is set to 1.5 m, with the main beam horizontally emitted. The distance from the horn to the corner of the L-shaped scenario is 3 m. The operating frequency of the transmitting horn is 5.8 GHz, with an equivalent isotropic radiated power (EIRP) set to 20 dBm. As shown in Fig. 7h, an RIS is installed at the corner of the corridor at a height of 1.5 m. The calculated EIRP of the RIS is approximately 10 dBm. The power distribution of the NLOS part analyzed is shown in Fig. 7f. Notably, installing an RIS at the corner of the corridor can enhance the power density on the NLOS side. Moreover, the power density improvement is most significant when the RIS deflection angle is 60$^{\circ }$. Compared to no RIS, the power density is increased by approximately 8 dB on average after installing the RIS. When the RIS deflection angle is 50$^{\circ }$, the power density is increased by approximately 6 dB on average. When the RIS deflection angle is 45$^{\circ }$, the power density is increased by approximately 3 dB on average. The best power density improvement at larger angles is due to fewer reflections of the electromagnetic waves. Additionally, to further enhance the blind-spot coverage, the aperture of the RIS can be increased. Figure 7g–j show the power distribution in the corridor without an RIS and with RIS deflection angles of 45$^{\circ }$, 50$^{\circ }$ and 60$^{\circ }$, respectively. It is evident that installing an RIS significantly improves the power density in the NLOS corridor.

**Figure 8.**
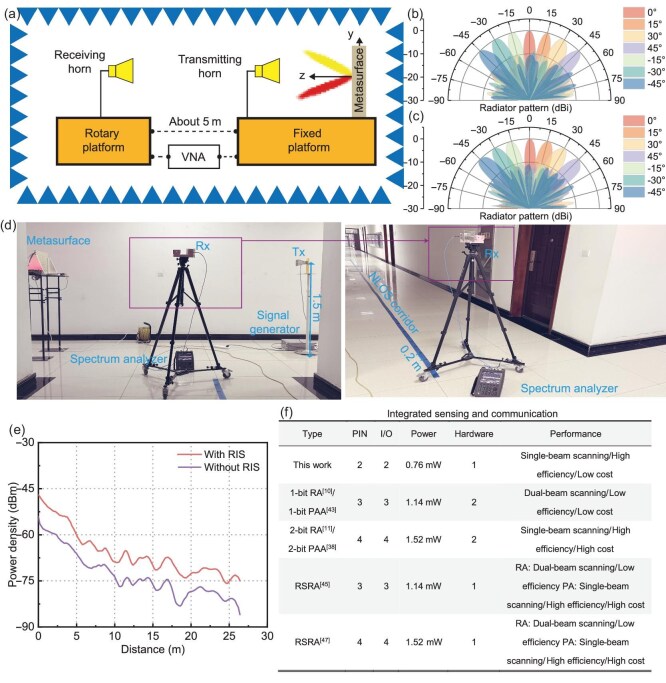
NLOS wireless communication experiment. (a) Test setup for the scattering mode. (b, c) Scattering mode test results at 5.8 GHz: (b) E plane and (c) H plane. (d) Power density testing in the L-shaped corridor. (e) Power density test results in the L-shaped corridor. (f) Cost and performance comparison of an RIS in integrated communication and sensing scenarios (RA, reflectarray: PPA, programmable phased array; RSRA, radiation-scattering reconfigurable array.

The beam-scanning characteristics of the proposed RIS in scattering mode were tested in the scenario shown in Fig. 8a. The E-plane and H-plane beam-scanning results at 5.8 GHz are shown in Fig. 8b, c, respectively. Single-beam scanning is achieved within the $\pm 45^{\circ }$ beam-scanning range, with a side-lobe level greater than or equal to 10 dB. These results are similar to the simulated characteristics. Test results for other frequencies are provided in Fig. S18. The blind-spot coverage effect of the RIS was tested in the L-shaped corridor shown in Fig. 8d. The selected scenario is an L-shaped corridor in a teaching building. The actual test scenario was set up according to the settings in the simulation. The heights of the metasurface, transmitting horn and receiving horn were all set to 1.5 m. The sampling interval was 0.2 m. A signal source was used to transmit a 5.8-GHz signal with an output power of 10 dBm. A spectrum analyzer was used to receive the signal. The RIS deflection angle is 60$^{\circ }$. The power density on the NLOS side of the corridor with and without the RIS is shown in Fig. 8e. The simulation and test results show consistent characteristics. Compared to no RIS, the installation of an RIS increased the power density by approximately 7 dB. Therefore, the proposed RIS can be used to enhance the power density in communication blind spots. Materials and methods are presented in Section S15.

### Performance comparison

Unlike conventional RIS designs, the proposed radiation-scattering RIS integrates both radiation and scattering modes within a single hardware platform. This achieves significant savings in physical space and cost while ensuring multifunctionality across diverse application scenarios. The proposed RIS applications can be categorized into three scenarios: LOS wireless communication, NLOS wireless communication, and integrated communication and sensing (which merges LOS and NLOS communication). Figure 8f and Tables S10–S11 present performance and cost analyses comparing the proposed RIS with existing RIS technologies across these scenarios. While the proposed RIS and other solutions show advantages in LOS and NLOS wireless communication, the proposed RIS stands out as the optimal low-cost design in integrated communication and sensing, comprehensively outperforming alternatives in cost-effectiveness.

For the integrated communication and sensing scenario, Fig. 8f shows the cost and performance comparison with existing studies. This scenario integrates the aforementioned wireless communication and NLOS wireless communication. In this scenario, the RIS can transmit signals autonomously or relay signals from other base stations. Figure 8f presents several designs for integrated communication and sensing. To achieve integrated communication and sensing, existing technologies can use a patchwork approach. For example, one can integrate a 1-bit reflectarray (RA) with a 1-bit programmable phased array (PAA), or a 2-bit RA with a 2-bit PAA. Figure 8f compares the designs in terms of the number of PIN diodes, number of I/O ports, hardware resources and performance. Hardware resources refer to the number of PIN diodes, number of I/O ports, energy, PCB cost, hardware system, etc., due to inconsistencies in the number of PIN diodes, I/O ports and energy loss across the designs. They are compared separately. Excluding the above factors, hardware resources involve several relatively uniform aspects, such as PCB area. Thus, they are combined into a single entry, and the multiples increased compared to the proposed RIS are recorded. As can be seen, the proposed RIS achieves the lowest cost with the fewest PIN diodes, I/O ports, energy loss and hardware resources when considering profile and efficiency. Thus, from the above three application scenarios, it can be concluded that the proposed RIS has a low cost. Although other RISs also have advantages in wireless communication and NLOS wireless communication, in integrated communication and sensing, the proposed RIS is the best low-cost design in all aspects.

## CONCLUSION

We propose an ingeniously designed low-cost all-in-one radiation-scattering RIS. The proposed radiation-scattering RIS integrates multiple functions, enabling a cost-effective phased array, and NLOS communication. The radiation-scattering meta-atom consists of a radiating patch and a 3-dB coupler. PIN diodes are loaded onto the 3-dB coupler to achieve 1-bit scattering and 1-bit radiation phase control. By loading capacitors of different values onto the radiating patch, four distinct initial phase designs are achieved. Arrays composed of these four types of radiation-scattering meta-atoms can suppress grating lobes caused by quantization errors in 1-bit scattering and radiation RISs. The 1-bit radiation design enables a cost-effective phased array, while the 1-bit scattering design supports NLOS communication. Moreover, when the two PIN diodes are turned off, the radiation-scattering RIS can perform WEH. A $12\times 12$ radiation-scattering RIS was fabricated to demonstrate its phased array, and NLOS blind-spot coverage capabilities. Moreover, compared to the limited existing radiation-scattering RIS implementations, we propose a comprehensive design framework. This framework employs a decoupled design approach for antenna elements and tunable circuits. Consequently, it enables the realization of radiation-scattering RISs with arbitrary polarization (e.g. circular, dual polarization) and phase solely by modifying antenna characteristics or tunable circuits. Therefore, under stringent hardware constraints and limited physical space, our radiation-scattering RIS provides multifunctional capabilities within a single platform. It can simultaneously function in wireless systems, and radar detection. These functionalities can be combined to enable more complex application scenarios.

The radiation-scattering design framework holds the potential to advance metasurface science towards deeper interdisciplinary integration. From a physical mechanism perspective, this establishes a new mechanism for investigating wavefront manipulation and electromagnetic cooperative stealth. Its underlying ‘functional layer–control layer’ decoupling architecture can be further integrated with emerging materials, such as phase-change materials. At the information system level, this method lays a hardware foundation for realizing a truly ‘intelligent electromagnetic environment’. In the future, this architecture could enable the development of environment-adaptive integrated sensing and communication systems, micro-base-station and relay-integrated systems, as well as self-powered sensing systems. This would facilitate the efficient multiplexing of multiple physical functions in complex scenarios. It will promote deeper convergence of information theory, electromagnetics and control science. Ultimately, it is expected to have a broad impact on 6G communications, the Internet of Things, intelligent stealth, and other related fields.

## Supplementary Material

nwaf470_Supplemental_File

## Data Availability

All data are available in the main text. Additional data related to this paper may be requested from the corresponding author.
