# Supplementary material for: Electromagnetic all-in-one radiation-scattering reconfigurable intelligent metasurface
Source: Natl Sci Rev. 2025 Nov 3;12(12):nwaf470. doi: 10.1093/nsr/nwaf470 (PMC12704106; doi:10.1093/nsr/nwaf470)
Supplement: nwaf470_Supplemental_File [file nwaf470_supplemental_file.pdf]

Supplementary Materials for

Electromagnetic All-in-One Radiation-Scattering Reconfigurable Intelligent Metasurface

Yajie Mu, Jiaqi Han, Hao Xue, Qiang Feng, Lingyun Niu, Haixia Liu, and Long Li

Corresponding author: Long Li, lilong@mail.xidian.edu.cn

The PDF file includes:

Sections S1 to S16
Figs. S1 to S19
Tables S1 to S12

Section S1: Meta-atom Design Principle

The key to the 1-bit radiation-scattering meta-atom design is the 3 dB coupler. The most critical aspect of the 3 dB coupler design is the incorporation of PIN diodes on both the through and coupled branches. The PIN diode model used is SMP1340. The PIN diodes are modeled as lumped elements in the simulation software. In the ON state, the equivalent circuit of the PIN diode is a series combination of a 0.8 Ω resistor and a 0.45 nH inductor. In the OFF state, it is equivalent to a series combination of a 0.21 pF capacitor and a 0.45 nH inductor. Figure S1a shows the S-parameters of port 2 of the 3 dB coupler when the PIN diodes are in states 00 and 11. Figure S1b shows the S-parameters of port 2 when the PIN diodes are in states 01 and 10. It can be observed that when the PIN diodes are in states 00 and 11, port 2 is well-matched. Moreover, the transmission phase difference between port 2 and ports 3 and 4 is 180°. When the PIN diodes are in states 01 and 10, port 2 exhibits total reflection. Moreover, at 5.8 GHz, the reflection phase at port 2 exhibits a 180° phase difference between these two states. Therefore, by incorporating PIN diodes on the through and coupled branches of the 3 dB coupler, 1-bit phase control of the radiation and scattering phases can be achieved.

Figure S1c illustrates the structure of a conventional 3 dB coupler. The conventional 3 dB coupler does not incorporate PIN diodes. Port 1 serves as the input port, while port 2 is the isolated port. Ports 3 and 4 are the through and coupled ports, respectively. Fig. S1d shows the transmission phase from port 1 to ports 3 and 4. The transmission phase difference is 90°, which is characteristic of a 3 dB coupler. Fig. S1e presents the matching characteristics of the four ports of the 3 dB coupler. The coupler is well-matched within the frequency range of 5.3-6.3 GHz. Figure S1f shows the transmission coefficients from port 1 to ports 2, 3, and 4. It can be seen that $|S_{13}|$ and $|S_{14}|$ are the transmission coefficients to the through and coupled ports, with a transmission coefficient of approximately 3 dB. In contrast, $|S_{12}|$ is the transmission coefficient to the isolated port, which is nearly zero. Thus, the 3 dB coupler we used functions as a conventional 3 dB coupler in the absence of PIN diodes. By incorporating two PIN diodes into the 3 dB coupler, we can achieve 1-bit phase control of the radiation and scattering phases. Therefore, our design represents the most concise and cost-effective approach for a 1-bit radiating and scattering metasurface. Table S1 lists the structural parameters of the 3 dB coupler. Table S2 provides the structural parameters corresponding to the four types of meta-atoms with different initial phases. Tables S3 and S4

present the initial phases and state switching of the four meta-atoms in both radiation and scattering modes.

Figure S2 presents the matching and radiation phases of the four meta-atoms after removing the 3 dB coupler. It can be observed that the four meta-atoms operate within the frequency range of 5.75-5.85 GHz. Moreover, the radiation phases of the four meta-atoms exhibit different initial phases. These initial phases are approximately 0°, 45°, 90°, and 135°, respectively. Therefore, the key aspects of our designed radiation-scattering meta-atoms with initial phases are twofold. The first aspect involves achieving 1-bit phase control of the radiation and scattering phases using a 3 dB coupler loaded with PIN diodes. The second aspect is the design of patch antennas with different initial phases. By integrating these two components, we can achieve a 1-bit radiation-scattering meta-atom design with different initial phases. Furthermore, our design approach can be extended to the design of circularly and dual-polarized 1-bit radiation-scattering meta-atoms. Our design method addresses the gap in the design of circularly and dual-polarized 1-bit radiation-scattering meta-atoms. Additionally, it represents the simplest method for designing 1-bit radiation-scattering meta-atoms.

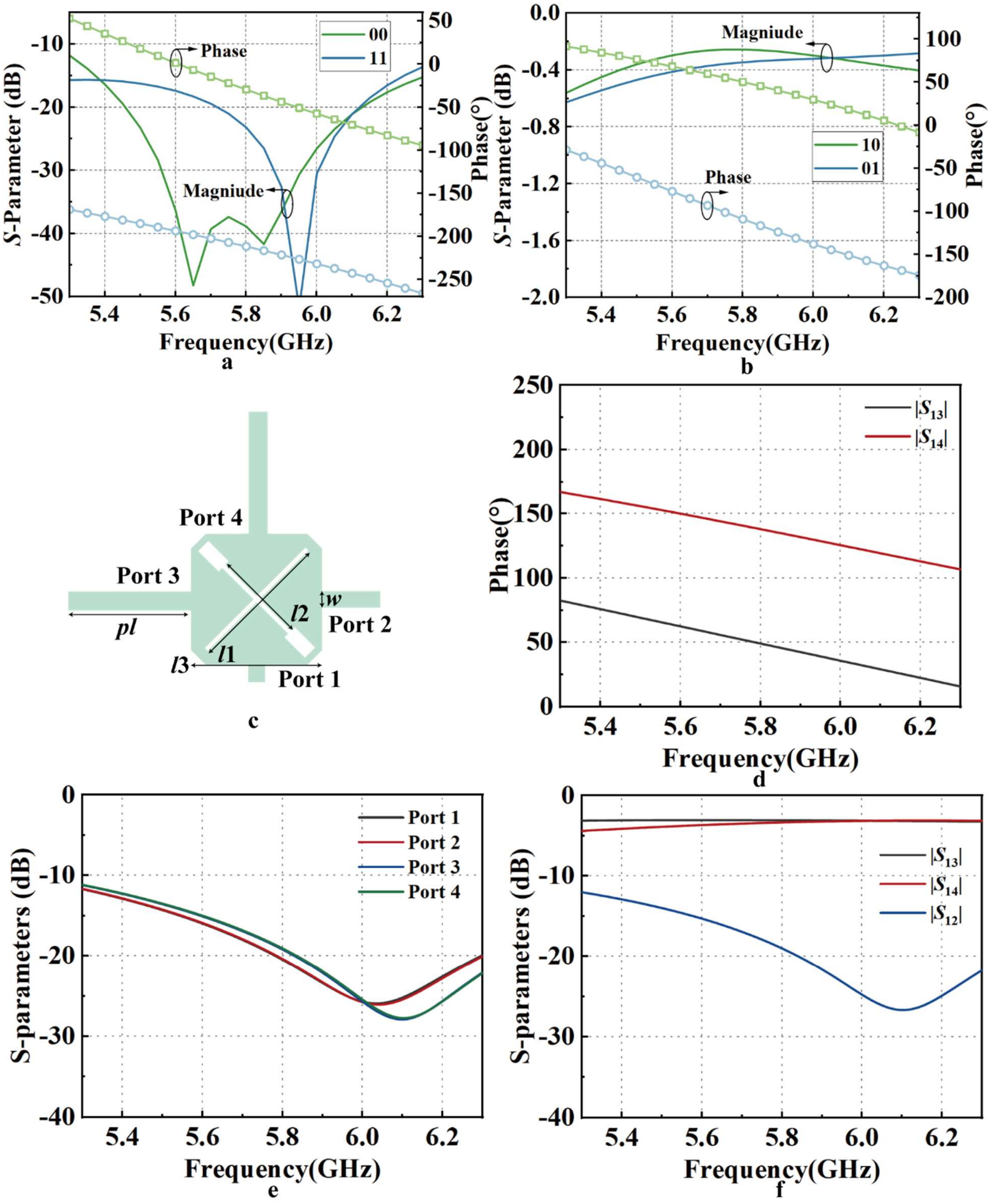

Fig. S1. 3 dB coupler performance. *S*-parameters of the 3dB coupler port 2 with PIN in (a) match mode and (b) reflection mode. (c) 3dB coupler without PIN. (d) Phase, (e) reflection coefficient, and (f) transmission coefficient of 3dB coupler without a PIN.

TABLE S1

Geometric parameters of coupler

Parameters	Value	Parameters	Value	Parameters	Value
pl	8 mm	*l*1	9 mm	*l*2	7 mm
*l*3	8 mm	*w*	1.1 mm		

TABLE S2

Geometric parameters of meta-atom

Parameters	*pxi*	*pxo*	*pyi*	*pyo*	C
Unit 1	4.8 mm	11 mm	7 mm	11.6 mm	0 pF
Unit 2	3.5 mm	11 mm	7 mm	12.1 mm	0.1 pF
Unit 3	3.5 mm	11 mm	7 mm	12.5 mm	0.2 pF
Unit 4	8.8 mm	10 mm	7 mm	10.7 mm	0.5 pF

TABLE S3

Meta-atom for radiation mode

Parameters	*PIN-1*	*PIN-2*	*State*	*Bit*	*Initial phase*
Unit 1	ON	ON	11	1-bit	0°
	OFF	OFF	00		
Unit 2	ON	ON	11	1-bit	45°
	OFF	OFF	00		
Unit 3	ON	ON	11	1-bit	90°
	OFF	OFF	00		
Unit 4	ON	ON	11	1-bit	135°
	OFF	OFF	00		

TABLE S4

Meta-atom for scattering mode

Parameters	*PIN-1*	*PIN-2*	*State*	*Bit*	*Initial phase*
Unit 1	ON	OFF	10	1-bit	90°
	OFF	ON	01		
Unit 2	ON	OFF	10	1-bit	180°
	OFF	ON	01		
Unit 3	ON	OFF	10	1-bit	270°
	OFF	ON	01		
Unit 4	ON	OFF	10	1-bit	0°
	OFF	ON	01		

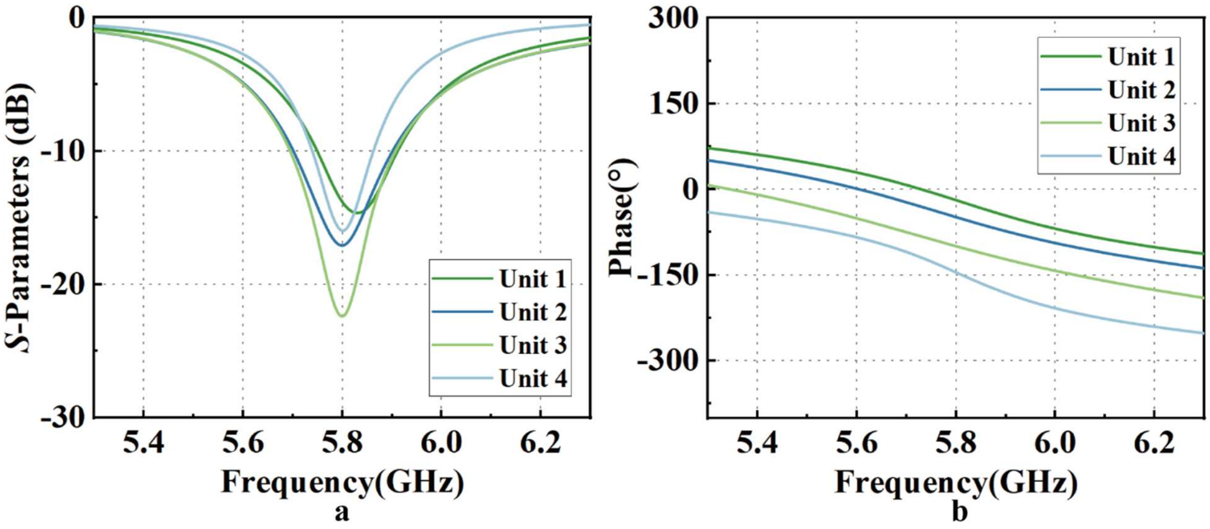

Fig. S2. *S*-parameters of the U-slot patch antenna when no 3 dB coupler is connected. (a) Reflection coefficient. (b) Radiation phase.

Section S2: Radiation-Stealth Meta-Atom Parameter Analysis

The 1-bit radiation-scattering meta-atom is primarily designed to control the 1-bit phase using PIN diodes loaded onto the 3 dB coupler. The PIN diodes are specifically loaded onto the through and coupled branches of the 3 dB coupler. Consequently, the lengths of the through and coupled branches are the primary factors influencing the phase of the designed meta-atom. Therefore, we subsequently analyze the influence of the through and coupled branch lengths (denoted as *pl*) on the S-parameters of the radiation and scattering modes. For brevity, we present only the analysis results for Unit 1. Units 2, 3, and 4 exhibit similar characteristics.

The *S*-parameters of the radiation mode for different *pl* values are shown in Figs. S3 (a to d). It can be observed that changes in *pl* have minimal impact on the matching performance of the radiation mode. That is, the operating bandwidth of the radiation mode remains at 5.75-5.85 GHz for different *pl* values. However, different *pl* values significantly affect the radiation phase. Fig. S3b shows the radiation phase in the 00 state. It can be seen that changes in *pl* have minimal effect on the radiation phase in the 00 state, with almost no change in the phase of the radiation mode. Fig. S3d shows the radiation phase in the 11 state. Notably, *pl* has a significant impact on the radiation phase in the 11 state. When *pl* is 6 mm, the radiation phases in the 00 and 11 states are -22° and -162°, respectively. However, the phase difference between these two states is only 140°, which does not meet the 1-bit phase requirement. However, as *pl* increases from 6 mm to 8 mm, the radiation phases in the 00 and 11 states are -29° and -202°, respectively. At this point, the phase

difference between the two states is 173°, which meets the 1-bit phase requirement. Therefore, as pl increases, the phase difference between the 00 and 11 states increases.

The S-parameters of the scattering mode for different *pl* values are shown in Figs. S3 (e to h). Variations in *pl* have a minimal effect on the resonant frequencies of the scattering mode. Specifically, for different *pl* values, the resonant frequencies of the scattering mode in the 01 and 10 states are approximately 5.7 GHz and 5.9 GHz, respectively. However, as *pl* increases, the resonant frequencies of the 01 and 10 states shift slowly towards lower frequencies. This results in a slight variation in the scattering phase. Overall, as *pl* increases from 6 mm to 8 mm, the phase differences between the 01 and 10 states are 161.8°, 163.9°, 166.6°, 169.0°, and 171.7°, respectively. Although *pl* has a minimal impact on the scattering mode, the 1-bit phase characteristics improve as *pl* increases.

Therefore, based on the analysis of the aforementioned parameters, we can draw a conclusion. That is, as *pl* increases, the phase difference between the two states in both radiation and scattering modes becomes larger. Moreover, the increase in *pl* has a minimal effect on the amplitude of radiation and scattering. Therefore, we can optimize the *pl* value to control the phase characteristics of the radiation-scattering meta-atom.

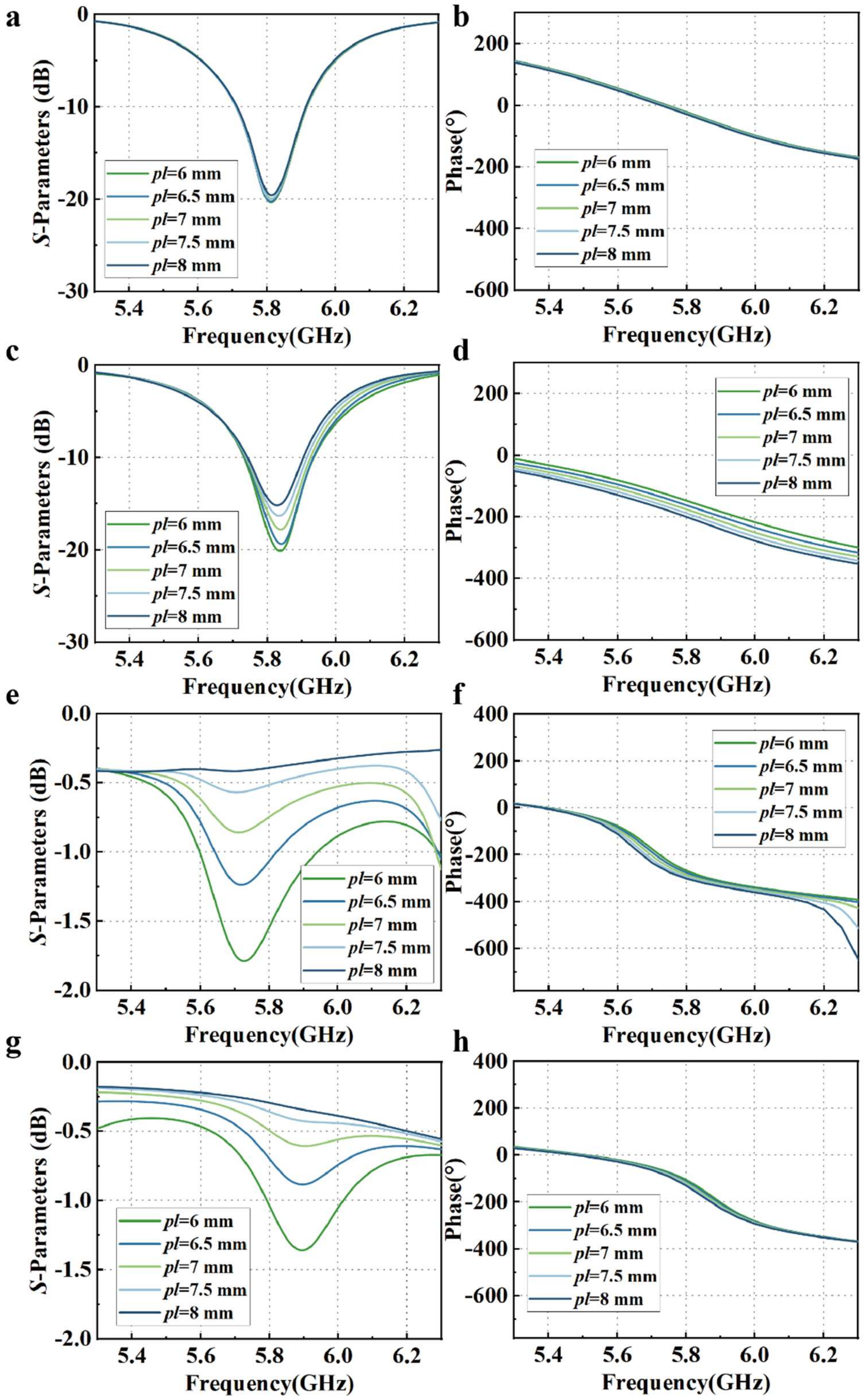

Fig. S3. Parametric analysis. (a) Magnitude, and (b) phase in radiation mode with 00 state. (c) Magnitude, and (d) phase in radiation mode with 11 state. (e) Magnitude, and (f) phase in scattering mode with 01 state. (g) Magnitude, and (h) phase in scattering mode with 10 state.

Section S3: Effect of Closed-State Resistance on Mate-Atom

The closed-state resistance of PIN diodes influences the radiation and scattering characteristics. In radiation mode, the PIN diode states are 00 and 11, with both diodes closed in state 11. The *S*-parameters and gain are illustrated in Figs. S4a-c. As the resistance increases from 0-6 Ω, the transmission coefficient from the lumped port to the Floquet port at 5.8 GHz decreases from -0.78 dB to -2.86 dB. However, resistance variations do not affect the matching characteristics of the meta-atom. Figure S4d shows the unit gain. As the resistance increases from 0-6 Ω, the gain decreases from 4.39 dBi to 2.18 dBi. Another state in radiation mode is 00, where both PIN diodes are open, making it unaffected by the closed-state resistance of the PIN diode. Similarly, in scattering mode, the two states of the PIN diodes for the meta-atom are 01 and 10. Figures S4e-f illustrates the reflection coefficients for states 01 and 10. It can be observed that as the resistance increases from 0-6 Ω, the reflection loss increases. For the 01 state, the reflection coefficient at 5.8 GHz decreases from -0.24 dB to -1.22 dB. For the 10 state, the reflection coefficient at 5.8 GHz decreases from -0.15 dB to -1.13 dB. Figures S4g-h shows the reflection phases for states 01 and 10. Notably, the reflection phase within the band is not affected by the resistance of the PIN diode. Therefore, the closed-state resistance of the PIN diode significantly impacts the performance of the meta-atom's radiation and scattering modes by directly affecting its gain and loss, leading to a reduction in the array's aperture efficiency. In practical testing, it is essential to ensure that the PIN diode is fully conductive to prevent high-resistance states under low current. Furthermore, dynamically adjusting the FPGA control characteristics allows modulation of the PIN diode's resistance, enabling amplitude modulation. Moreover, selecting PIN diodes with lower closed-state resistance during component selection helps enhance overall efficiency.

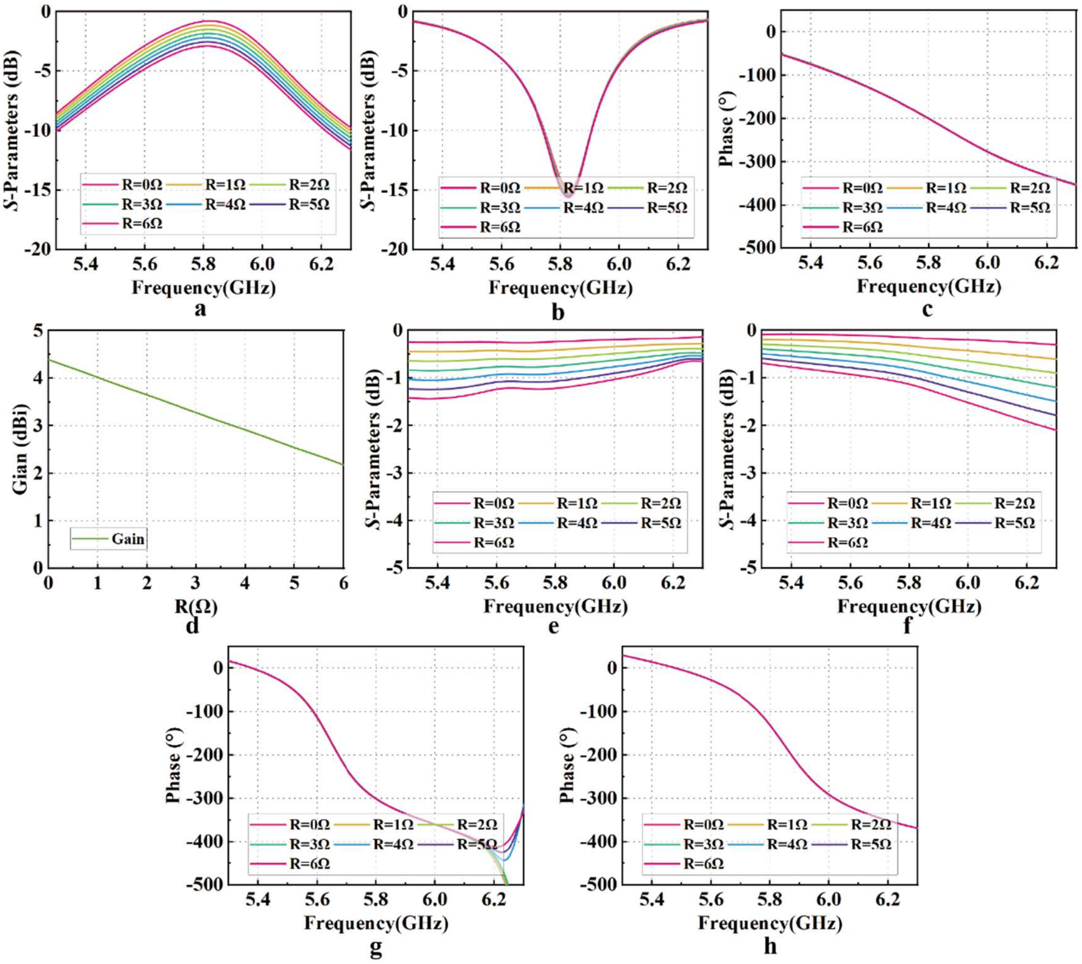

Fig. S4 Effect of closed-state resistance on the mate-atom. (a) Transmission coefficient, (b) reflection coefficient, (c) transmission phase, and (d) gain of meta-atom under 11 state of radiation mode. (e) 01, and (f) 10 states reflection coefficient of meta-atom under scattering mode. (g) 01, and (h) 10 states reflection phase of meta-atom under scattering mode.

Section S4: LHCP Radiation-Scattering Meta-Atom Design

The compatibility of designing circularly polarized radiation-scattering meta-atoms using a frontend to backend decoupling framework significantly enhances the universality and impact of our work. Figure S5 illustrates the structure and design process of the circularly polarized radiation-scattering meta-atom. Figure S5a shows the left hand circular polarization (LHCP) radiation-scattering meta-atom designed using the decoupling framework. The backend circuit adopts the design of a linearly polarized radiation-scattering meta-atom, with parameters and PIN diode states consistent with linear polarization. The only difference is the frontend receiving antenna, which is an LHCP antenna. Its reflection coefficient and axial ratio (AR) characteristics are shown in Figs. S5b-c. Its -10 dB matching bandwidth is 5.63-6.08 GHz, and the bandwidth

with AR less than 3 is 5.75-5.86 GHz. The corresponding unit radiation pattern in the xoz plane is shown in Fig. S5d. The gain is 4.35 dBi, and the corresponding right hand circular polarization (RHCP) amplitude is -23.6 dBi. Thus, the isolation between LHCP and RHCP is 27.95 dB. The above data indicate that the designed LHCP antenna has good characteristics. The designed circularly polarized antenna port is connected to the backend circuit, as shown in Fig. S5a. In radiation mode, the reflection coefficients, AR, and phases for states 00 and 11 are shown in Figs. S5e-g. It can be seen that the matching bandwidth is 5.62-6.16 GHz. The AR of states 00 and 11 completely overlap, and the AR is less than 3 in 5.75-5.86 GHz. This indicates that the radiation mode has circular polarization characteristics in 5.75-5.86 GHz. Figure S5g shows the radiation phases for states 00 and 11. It can be seen that stable 1-bit phase characteristics are maintained across the entire bandwidth. Figures S5h-i illustrate the scattering mode characteristics of the meta-atom. Figure S5h shows the reflection coefficient. It can be seen that there is a certain frequency offset in the reflection coefficients for states 01 and 10. Among them, the common bandwidth with RHCP less than -10 dB is 5.73-5.89 GHz. The LHCP reflection coefficients for states 01 and 10 are shown in Figure S5i. They exhibit stable 1-bit phase characteristics in the 5.73-5.89 GHz range. Furthermore, the bandwidths of both radiation and scattering modes are closely related to the AR characteristics. Therefore, to enhance the bandwidth of circularly polarized radiation-scattering meta-atoms, it is necessary to increase the AR bandwidth when designing circularly polarized antennas. Additionally, although it is elliptically polarized outside the AR bandwidth, it still exhibits 1-bit phase characteristics in both radiation and scattering modes. Thus, it can control elliptical polarization outside the bandwidth. Through the example of circularly polarized radiation-scattering meta-atoms, the flexibility of our decoupling design framework is demonstrated.

Therefore, by changing the polarization of the frontend receiving antenna, radiation-scattering meta-atoms with arbitrary polarization can be obtained. For dual-polarized radiation-scattering meta-atoms, only a dual-polarized antenna backend connected to two circuits is required. The frontend to backend decoupling design not only enables arbitrary polarization design but also allows for arbitrary initial phase and amplitude design. This can be achieved by designing the frontend receiving antenna. Consequently, the radiation-scattering meta-atom design we propose has strong compatibility and addresses the challenges in designing radiation-scattering meta-atoms with arbitrary polarization.

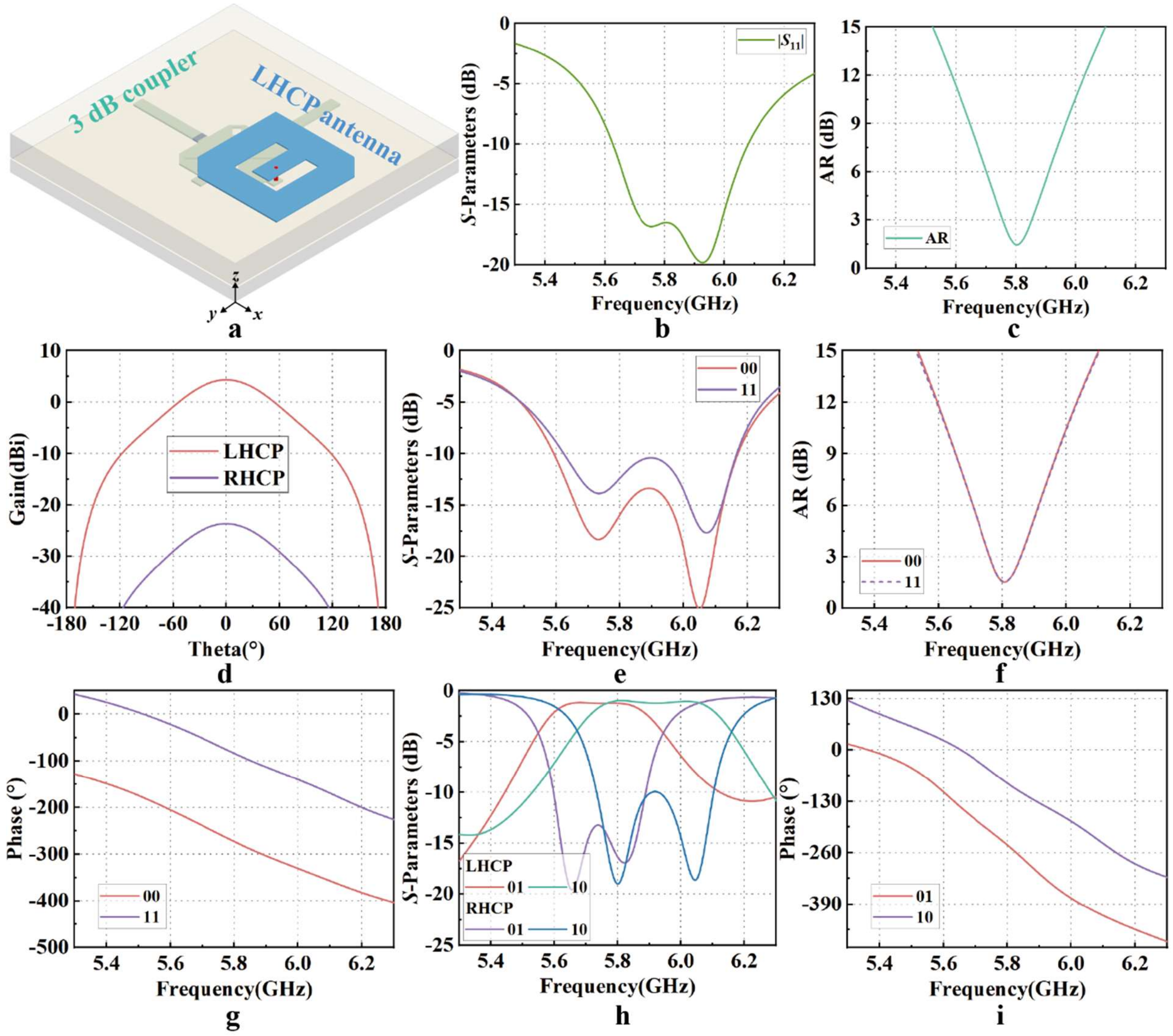

Fig. S5 LHCP radiation-scattering meta-atom design. (a) LHCP meta-atom structure. (b) Reflection coefficient, (c) AR, and (d) pattern of LHCP antenna. (e) Reflection coefficient, (f) AR, and (g) phase of LHCP meta-atom in radiation mode. (h) S-parameter, and (i) phase of LHCP meta-atom in scattering mode.

Section S5: Phase-Continuous Control Radiation-Scattering Meta-Atom Design

The use of a frontend-backend decoupling framework design for phase-continuous control radiation-scattering meta-atoms greatly improves the compatibility, thus enhancing the universality and impact of our work. Figure S6a illustrates the structure diagram of the phase-continuous control radiation-scattering meta-atom. The meta-atom is observed to remain composed of an antenna and a 3 dB coupler. The only difference lies in the types and numbers of diodes integrated into the 3 dB coupler. A varactor diode is loaded on the through and coupled stubs of the 3 dB coupler, respectively. The model of the varactor used in this design is Skyworks

SMV1430 whose junction capacitance ranges from 0.31 to 1.24 pF as reverse voltage changes from 30 to 0 V. Package series inductance is 0.45 nH. Continuous phase modulation of radiation and scattering is achieved by tuning varactor-1 and -2, which are kept in identical states. Radiation and scattering modes are switched by integrating a PIN-3 diode at Port 1. When the PIN-3 diode is closed, the meta-atom operates in radiation mode; otherwise, it operates in scattering mode. Additionally, the antenna remains linearly polarized. However, two PIN diodes are loaded onto the antenna to achieve 1-bit radiation phase modulation. This is because the 3 dB coupler can only achieve continuous modulation of radiation phase within 0-180° and scattering phase within 0-360°. Thus, the 3 dB coupler combined with the antenna enables continuous modulation of radiation phase across 0-360°. Essentially, the type of antenna remains unchanged. The operational states of the meta-atom with continuous phase modulation are presented in Table S5. Figures S6b-d show the S-parameters for radiation mode. When the capacitance of the varactor diode is tuned from 0.31 pF to 1.21 pF, the reflection coefficient of State 1 remains below -10 dB at 5.77-5.88 GHz. The reflection coefficient of State 2 exhibits similar behavior and is omitted. Figure S6c shows the radiation phase of State 1. When the capacitance of the varactor diode is tuned from 1.21 pF to 0.31 pF, the radiation phase varies between 0° and 180°. Figure S6d shows the radiation phase of State 2. When the capacitance of the varactor diode is tuned from 1.21 pF to 0.31 pF, the radiation phase varies between 180° and 360°. Thus, continuous modulation of radiation phase across 0-360° is achieved through the above tuning. Figures S6e-g show the S-parameters for scattering mode. When the capacitance of the varactor diode is tuned from 0.31 pF to 1.21 pF, the reflection coefficient of State 3 exceeds -3.6 dB at 5.8 GHz. The reflection coefficient of State 4 exhibits similar behavior and is omitted. Figure S6f shows the scattering phase of State 3. When the capacitance of the varactor diode is tuned from 1.21 pF to 0.31 pF, the radiation phase varies between 0° and 360°. Figure S6g shows the radiation phase of State 4. When the capacitance of the varactor diode is tuned from 1.21 pF to 0.31 pF, the radiation phase varies between 0° and 360°. Thus, continuous modulation of scattering phase across 0–360° is achieved through the above tuning. Through an example of a radiation-scattering meta-atom with continuous phase modulation, the flexibility of our decoupling design framework is demonstrated. Consequently, the proposed radiation-scattering meta-atom design exhibits strong compatibility, addressing key challenges in designing radiation-scattering meta-atoms with arbitrary polarization and continuous phase modulation.

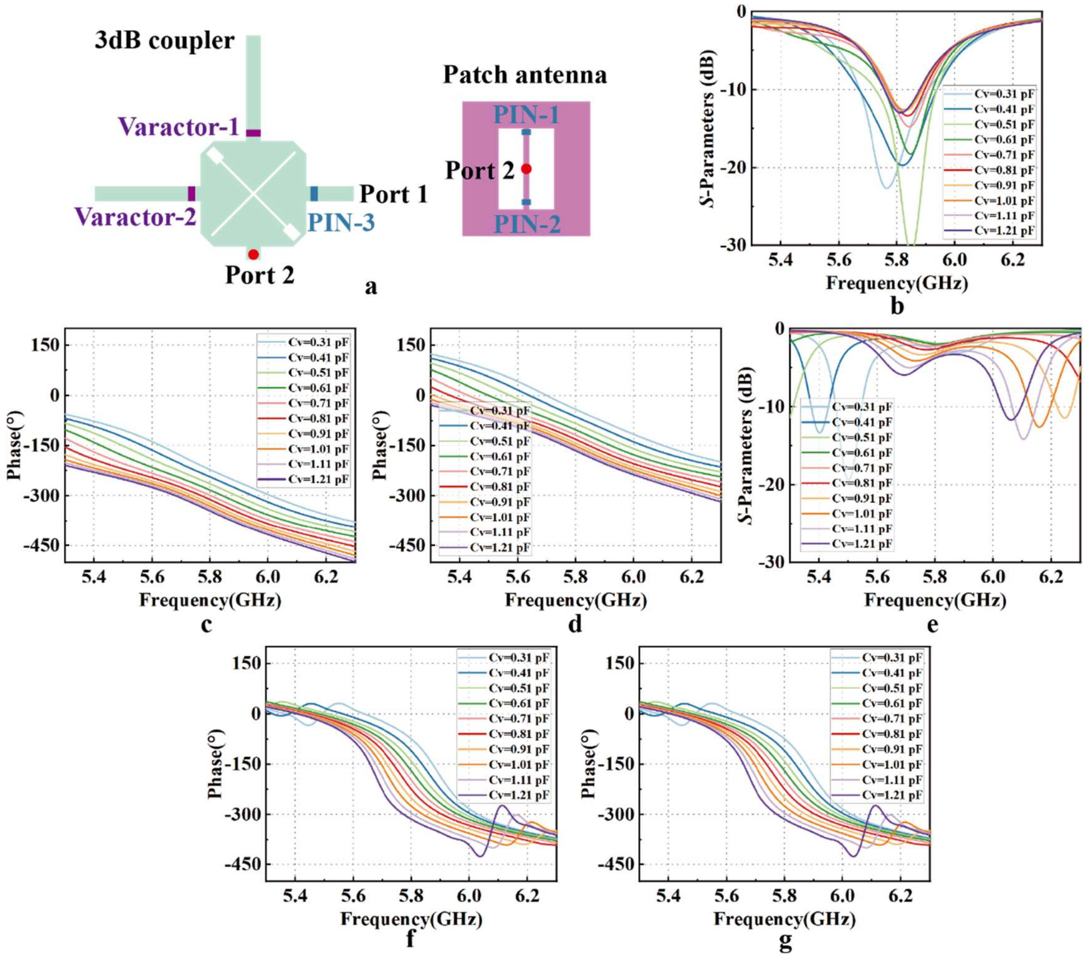

Fig. S6 Continuous phase control radiation-scattering meta-atom. (a) Meta-atom structure. (b) Reflection coefficient in State 1, (c) phase in State 1. and (d) phase in State 2 under radiation mode. (e) Reflection coefficient in State 3, (f) phase in State 3. and (g) phase in State 4 under scattering mode.

TABLE S5

Continuous phase control radiation-scattering meta-atom

Mode	*PIN-1*	*PIN-2*	*PIN-3*	*Varactor-1*	*Varactor-2*	*Phase*	*State*
Radiation mode	ON	OFF	ON	0.31-1.21 pF	0.31-1.21 pF	0°-180°	State 1
	OFF	ON	ON	0.31-1.21 pF	0.31-1.21 pF	180°-360°	State 2
Scattering mode	ON	OFF	OFF	0.31-1.21 pF	0.31-1.21 pF	0°-360°	State 3
	OFF	ON	OFF	0.31-1.21 pF	0.31-1.21 pF	0°-360°	State 4

Section S6: Initial Phase Design Principle

Figures S7a-b shows the coding and beam patterns of RIS at 1-bit quantization with (30°, 0°) without initial phase. It can be observed that the forward and backward phase gradients of the coding are symmetric and both 180°. This consequently results in dual-beam scanning. To suppress the dual-beam caused by 1-bit quantization, Fig. S7c introduces a set of initial phases randomly distributed within [0°, 360°]. Figure S7d illustrates the initial phases and their corresponding counts. It can be seen that there are 123 phases in total, with the same phase appearing at most three times. Figure S7e shows the coding of RIS at 1-bit quantization with (30°, 0°) under random initial phases. Notably, the forward and backward phase gradients of the coding are no longer symmetric under these conditions. The principle behind random initial phases suppressing grating lobes is disrupting the symmetric phase gradient caused by 1-bit quantization. The energy of the grating lobes is dispersed into different spatial regions. Figure S7f provides the beam characteristics under four types of random initial phases with (0°, 30°). It can be observed that single-beam scanning characteristics are achieved. However, the sidelobe characteristics are inconsistent, varying in quality. Therefore, optimization algorithms need to be introduced to optimize the initial phases to find better beam characteristics. Nevertheless, this undoubtedly increases the design complexity. Moreover, the current random approach results in the need for over 100 initial phases. This significantly increases the difficulty and complexity of initial phase design. Figure S7g shows the beam scanning results from 0° to 45° under unoptimized random initial phases. It can be observed that the sidelobe level exceeds 9.24 dB. To reduce the number of initial phases, quantization can be adopted, such as 3-bit, 2-bit, and 1-bit. Figures S7h-j correspond to beam scanning under random initial phases with 1-bit, 2-bit, and 3-bit quantization, respectively. Among them, random initial phases with 1-bit, 2-bit, and 3-bit quantization correspond to 2, 4, and 8 phases, respectively. This significantly reduces the difficulty and complexity of initial phase design. However, random initial phases with 1-bit quantization fail to suppress grating lobes and still result in dual-beam scanning. Random initial phases with 3-bit and 2-bit quantization can achieve single-beam scanning. However, the sidelobe level of beam scanning with random initial phases under 3-bit quantization is slightly better than that under 2-bit. Therefore, quantization can be employed to reduce the number of initial phases. Currently, 2-bit quantization is the minimum requirement for achieving single-beam scanning. Regardless of whether random initial phases are

quantized with 2-bit or 3-bit, optimization algorithms are still required to achieve better beam scanning performance. This is not the optimal method for initial phase design.

Therefore, building on the existing framework, we propose a method to obtain the initial phase without optimization algorithms. Under near-field horn excitation, the 1-bit RIS exhibits single-beam scanning characteristics. The wave from the near-field horn excitation generates a set of initial phases upon reaching the RIS. By compensating for the initial phases and the phase required for beam deflection, the RIS achieves superior single-beam scanning. Similarly, for plane waves incident on the RIS, the initial phase is 0°. Without an initial phase, dual-beam scanning occurs under 1-bit quantization. We predefine a set of initial phases similar to those from the near-field horn, except that these initial phases are provided by the RIS itself rather than the near-field horn. In such a case, the RIS can achieve single-beam scanning under 1-bit quantization. The underlying mechanism is that regardless of whether the excitation source is a near-field horn or a plane wave, as long as there is a set of initial phases on the RIS, compensating for these initial phases and the phase required for beam deflection can achieve single-beam scanning. The excellent beam-scanning characteristics of the reconfigurable reflectarray array under 1-bit quantization serve as the best evidence. Therefore, we use the initial phases introduced by the near-field horn to guide the acquisition of the optimal initial phases. The formula for the initial phase is as follows:

$$\varphi_f = k\sqrt{(x-x_0)^2+(y-y_0)^2+(z-z_0)^2} \quad (1)$$

Where (x, y, z) represents the position of each element. (x_0, y_0, z_0) is the position of the feed source, where $x_0 = 0$, $y_0 = 0$, $z_0 = \mathrm{F}$. The focal diameter ratio (FDR) is defined as F/D, where D is the size of the array. Therefore, optimizing the F/D ratio can achieve the optimal beam-scanning characteristics. Figure 7k shows the initial phase distribution when F/D=0.6. The 1-bit quantized beam scanning under plane wave incidence is shown in Fig. S7l. It can be seen that the sidelobe level exceeds 12.1 dB. Additionally, it possesses 19 types of initial phases. To reduce the complexity of initial phase design, the initial phases in Fig. S7k are subjected to 1-bit, 2-bit, and 3-bit quantization. Among them, random initial phases with 1-bit, 2-bit, and 3-bit quantization correspond to 2, 4, and 8 phases, respectively. This significantly reduces the difficulty and complexity of initial phase design. Figures S7m-o correspond to beam scanning under initial phases with 1-bit, 2-bit, and 3-bit quantization, respectively. However, initial phases with 1-bit quantization fail to suppress grating lobes and still result in dual-beam scanning. Initial phases

with 2-bit and 3-bit quantization can achieve single-beam scanning. The sidelobe level of beam scanning with 3-bit quantization is only slightly better than that with 2-bit quantization. While more initial phase types generally lead to better beam-scanning characteristics, considering the balance between initial phase design complexity and beam-scanning performance, 2-bit quantization is selected. Therefore, the above method avoids the need for optimization algorithms to obtain a set of good initial phases. Next, 2-bit initial phases can be realized by designing meta-atoms.

The proposed initial phases of radiation and scattering for the meta-atom are interrelated. Therefore, after the initial phase of the scattering mode is determined using the aforementioned method, the initial phase of the radiation mode is also defined. The initial phases of radiation are 0°, 45°, 90°, and 135°. Although the radiation initial phases are not strictly derived from Equation (1), they are still capable of suppressing grating lobes.

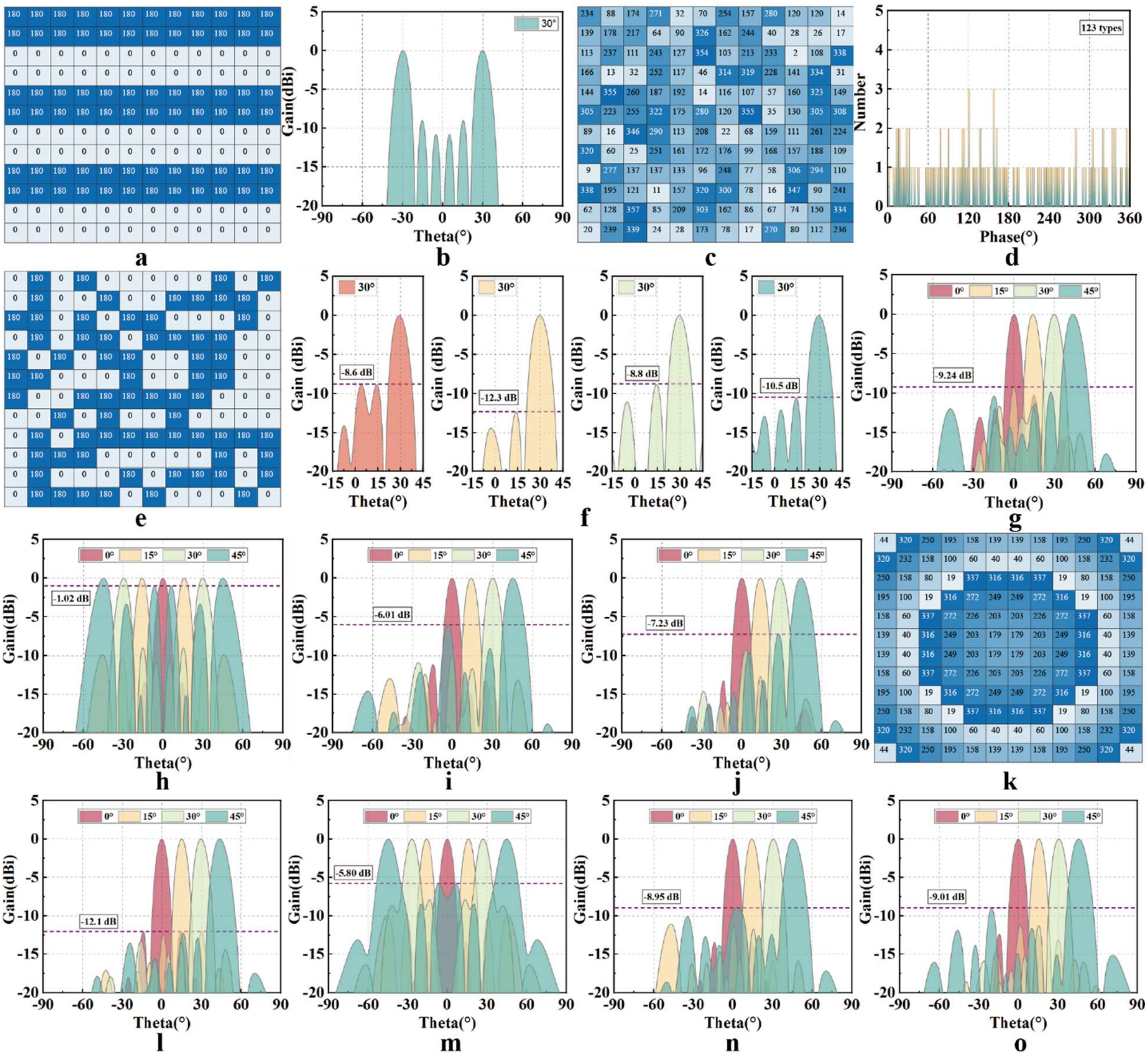

Fig. S7 Initial phase design principle. (a) (30°, 0°) coding, and (b) beam pattern under without initial phase. (c) Random initial phase, and (d) initial phase types. (e) (30°, 0°) coding, and (f) corresponding beam pattern under random initial phase. Beam pattern under random initial phase with (g) continues, (h) 1-bit, (i) 2-bit and (j) 3-bit quantization. (k) Initial phase based on near-field feeding mechanism. Corresponding beam pattern under initial phase with (l) continues, (m) 1-bit, (n) 2-bit and (o) 3-bit quantization.

Section S7: Overall Schematics of the Radiation-Stealth Metasurface

The layout of the fabricated array is shown in Fig. S8. It includes the top patch array, the metallic ground plane, the DC bias network, and the 3 dB coupler with its power combiner. Different layers are marked with different colors (purple, yellow, blue, and red). The substrate is represented in white. The circles around the array indicate the alignment holes.

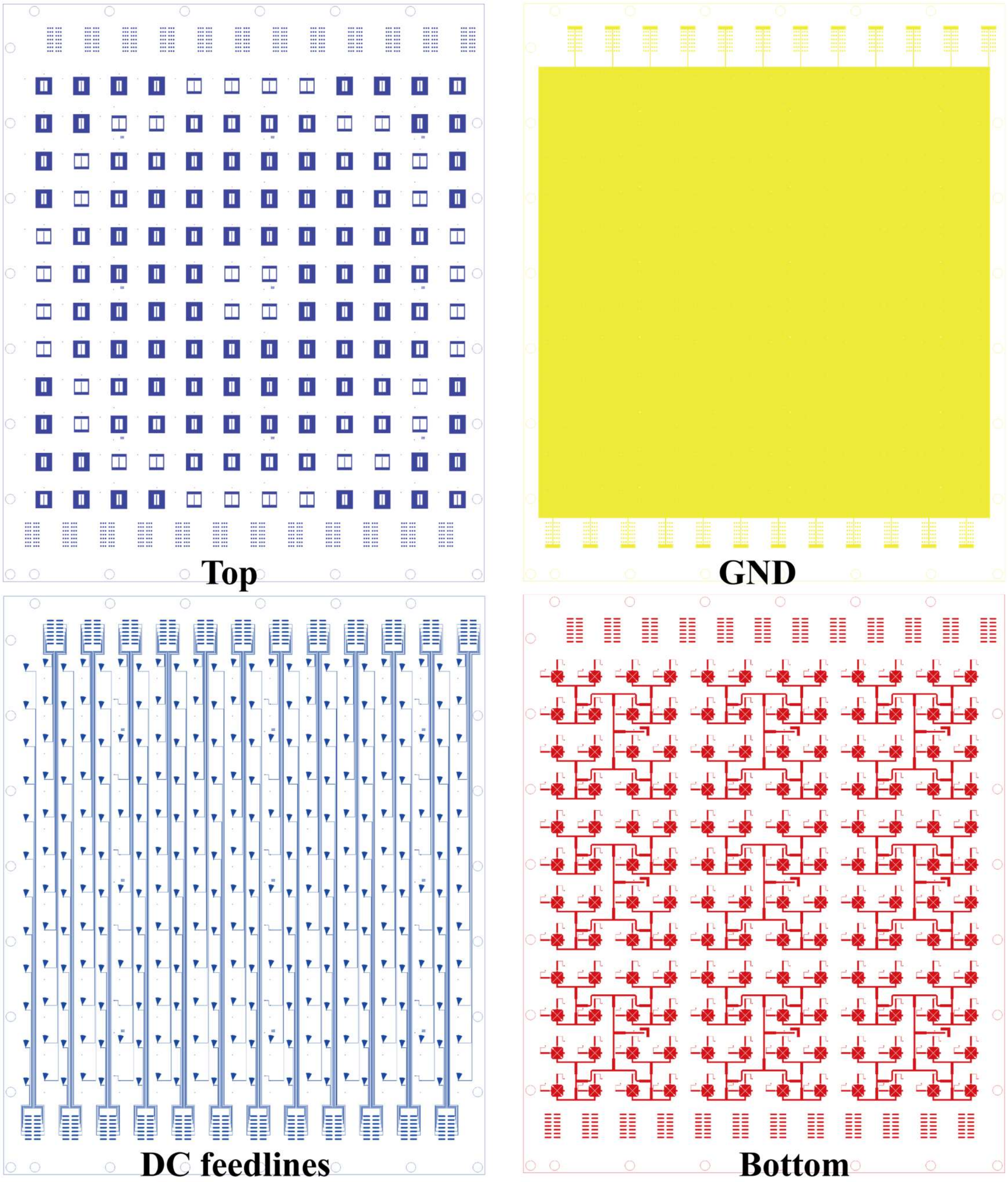

Fig. S8. Fabrication Drawings of Metasurface.

Section S8: Initial Phase Configuration of Metasurface Array

The initial phases of the 12×12 array obtained using the initial phase design method are shown in Fig. S9a. The initial phases obtained have a large number of distinct values. Designing metasurface unit cells based on such a large number of initial phases would not only be complex but also

difficult to realize. Therefore, we quantize the initial phases to 2-bit levels. The quantized initial phases are shown in Fig. S9b. After 2-bit quantization, only four initial phases remain, namely 0°, 90°, 180°, and 270°. This greatly simplifies the design complexity of the initial phases. Fig. S9c shows the arrangement of atoms corresponding to the four initial phases. It can be seen that the four types of meta-atoms are arranged in the array according to their respective initial phases.

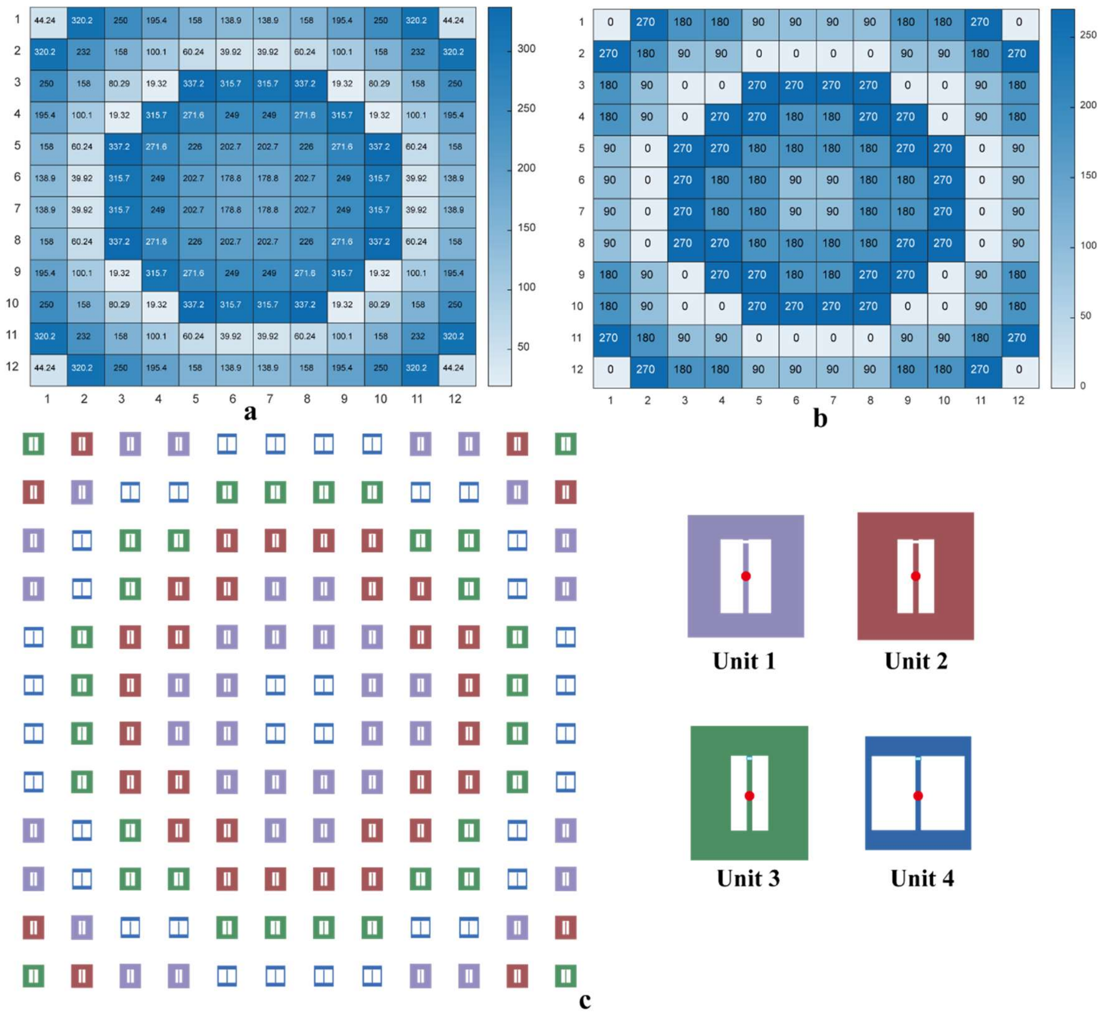

Fig. S9. Initial phase for RIS. (a) Initial phase distribution before quantization. (b) Initial phase distribution after 2-bit quantization. (c) Array topology corresponding to the initial phase distribution after 2-bit quantization.

Section S9: Beam Scanning in Radiation Mode

In the radiation mode, the beam-scanning results at other frequencies obtained through simulation are shown in Fig. S10. It can be observed that single-beam scanning of ±45° is achievable at both 5.75 GHz and 5.85 GHz. Single-beam scanning of ±45° is also achievable in both the E-plane and

H-plane. While the side-lobe level of beam scanning at large angles is slightly lower, the side-lobe level for other angles is greater than 10 dB. Therefore, the introduction of initial phases in the 1-bit scattering reconfigurable intelligent surface (RIS) can suppress grating lobes during beam scanning. Moreover, the quantized initial phases also effectively enhance the side-lobe level during beam scanning.

In the radiation mode, the beam-scanning results at other frequencies obtained through testing are shown in Fig. S11. It can be seen that single-beam scanning of ±45° is achievable at both 5.75 GHz and 5.85 GHz. These results are consistent with the simulation outcomes. The side-lobe level is slightly lower for large-angle beam scanning, while for other angles, the side-lobe level is greater than 10 dB.

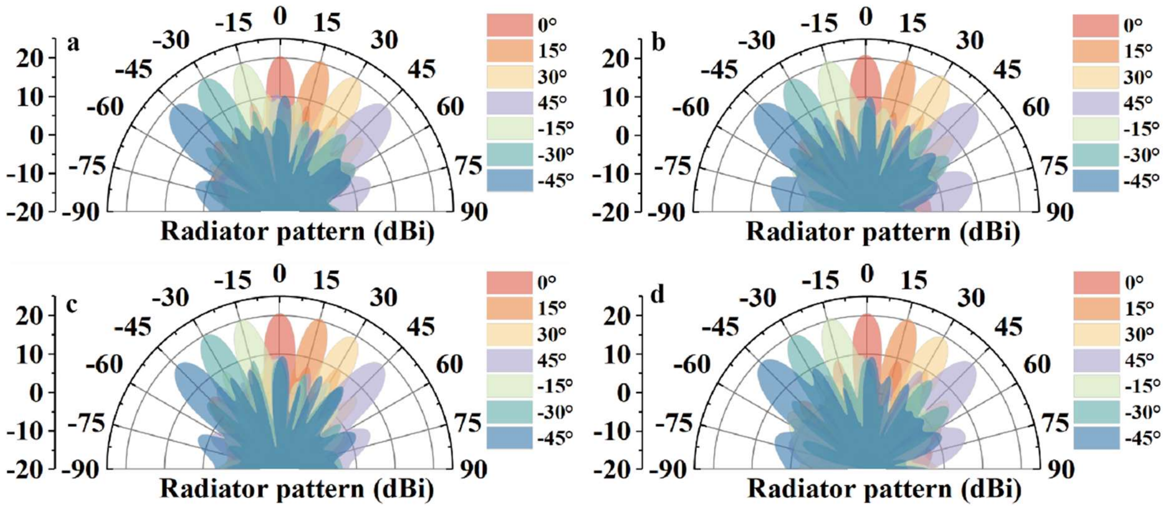

Fig. S10. Simulated beam scanning results. (a) 5.75 GHz for E-plane. (b) 5.75 GHz for H-plane. (c) 5.85 GHz for E-plane. (d) 5.85 GHz for H-plane.

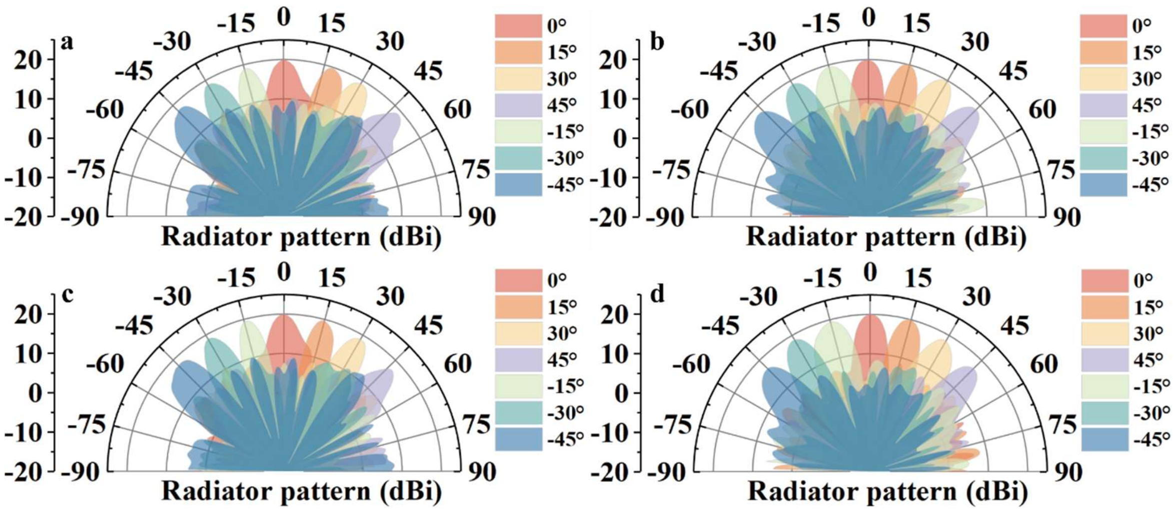

Fig. S11. Measured beam scanning results. (a) 5.75 GHz for E-plane. (b) 5.75 GHz for H-plane. (c) 5.85 GHz for E-plane. (d) 5.85 GHz for H-plane.

Section S10: Beam Scanning without Initial Phase in Radiation Mode

To highlight the advantages of initial phase design, we present the beam characteristics of RIS without initial phase. Figure S12 illustrate the beam-scanning characteristics of RIS in radiation mode. Figures S12a and b show the E-plane beam-scanning characteristics at positive angles without and with initial phase, respectively. It can be observed that the absence of initial phase results in dual-beam scanning. Moreover, the beam-scanning sidelobes from 0° to ±45° are significantly large, with the sidelobes at ±45° almost matching the main lobe. Specifically, the gains at 0°, ±15°, ±30°, and ±45° are 23.1 dBi, 20.5 dBi, 19.83 dBi, and 18.26 dBi, respectively. It is evident that the gain of the scanned beam drops rapidly. In contrast, with the initial phase, RIS achieves single-beam scanning. The sidelobes are maintained below 10.1 dB. The gains at 0°, 15°, 30°, and 45° are 21.33 dBi, 21.2 dBi, 19.92 dBi, and 18.80 dBi, respectively. Although the array's broadside gain is reduced with the initial phase, the scanned beam gain and sidelobe levels are superior to those without the initial phase. Figures S12c and d depict the H-plane beam-scanning characteristics at positive angles without and with initial phase, respectively. It can be observed that the H-plane exhibits similar characteristics. Therefore, the distinction between using and not using initial phases goes beyond merely suppressing grating lobes. With initial phases, the scanning gain of the array is enhanced, and sidelobe levels are reduced.

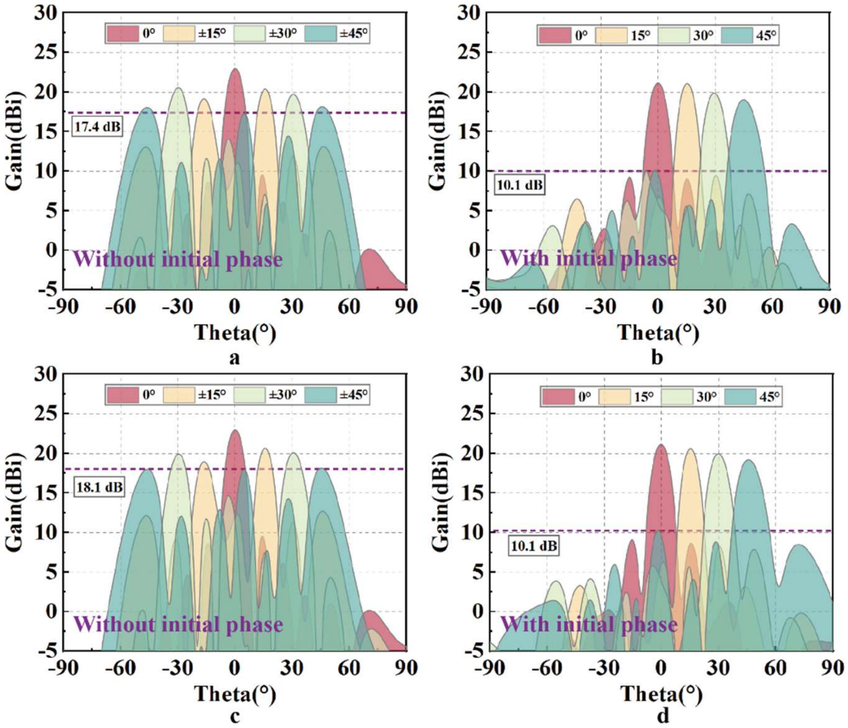

Fig. S12 Beam scanning in radiation mode. E-plane scanning (a) with, and (b) without initial phase. H-plane scanning (c) with, and (d) without initial phase.

Section S11: 1-to-9 Power Divider

To test the radiation characteristics of the 12×12 array, it is necessary to excite all nine RF ports simultaneously. Therefore, a 1-to-9 power divider was fabricated, as shown in Fig. S13. When operating in radiation mode, RIS is excited by feeding RF signals of equal amplitude and phase through 9 ports. However, during actual testing, we used a 1-to-9 power divider to excite the 9 RF ports. In both simulations and measurements, it is difficult to achieve exact equal amplitude and phase at the power divider ports. Therefore, it is necessary to analyze the impact of amplitude and phase errors in the 1-to-9 power divider on RIS characteristics. Figures S14a-c present the simulated performance of the 1-to-9 power divider. Figures S14d-f present the measured performance of the 1-to-9 power divider. Tables S6 and S7 present the relative amplitude and phase of the 9 ports of the power divider at 5.8 GHz for simulation and measurement, respectively. It can be seen that the maximum amplitude and phase errors of the 9 ports in simulation are 0.4 and 14.55°, respectively, while those in measurement are 0.54 and 19.91°, respectively. This indicates that the measured consistency of amplitude and phase at the power divider ports is slightly worse than that in simulation. As the 9 ports of the RIS are matched, the data of the 9 ports

from the simulated and measured power dividers can be incorporated into the RIS. The differences in beam characteristics compared to the ideal case can be analyzed. Additionally, Table S8 presents the signal conditions of the 9 ports when the port phases are aligned and the maximum amplitude error is 0.81. Table S9 presents the signal conditions when the port amplitudes are aligned and the maximum phase error is 74°. Through the above four feeding scenarios, the fault tolerance of RIS beam scanning is analyzed, as shown in Fig. S15. Only the beam scanning results for negative angles in the E-plane are presented for illustration, with similar results observed for other cases. Additionally, the beam characteristics under ideal conditions (with equal amplitude and aligned phase at all 9 ports) are provided for comparison.

Case 1 and Case 2 present the beam-scanning characteristics of RIS under simulated and measured data feeding conditions from the 1-to-9 power divider ports. It should be noted that the poor amplitude and phase consistency of the power divider leads to minor degradation in the gain and sidelobes of the 0° to -45° beams. Specifically, the gain degradation at 0° and -15° is approximately 1.35 dB and 0.73 dB, respectively. In contrast, the gain at -30° and -45° remains unaffected. The sidelobe degradation at 0°, -15°, -30°, and -45° is approximately 0.5 dB. Thus, the introduction of the 1-to-9 power divider leads to slight degradation in beam characteristics. However, the extent of degradation is minor and has a negligible impact on RIS performance. Case 3 and Case 4 represent the beam characteristics under conditions of worse amplitude and phase consistency compared to Cases 1 and 2. Poor amplitude consistency, analogous to amplitude tapering in phased arrays, reduces sidelobes but also causes gain degradation, particularly at 0° and -15°. Poor phase consistency affects both gain and sidelobes. Degradation is evident at 0° and -15° but not significant at -30° and -45°. While poor amplitude and phase consistency in the feeding signals of RIS ports can lead to beam gain and sidelobe degradation, the beam direction remains unaffected. Additionally, the 1-to-9 power divider exhibits much smaller amplitude and phase consistency errors compared to Cases 3 and 4, where gain and sidelobe degradation is approximately 2 dB. Therefore, the impact of phase and amplitude consistency errors in the 1-to-9 power divider on RIS performance is within an acceptable range.

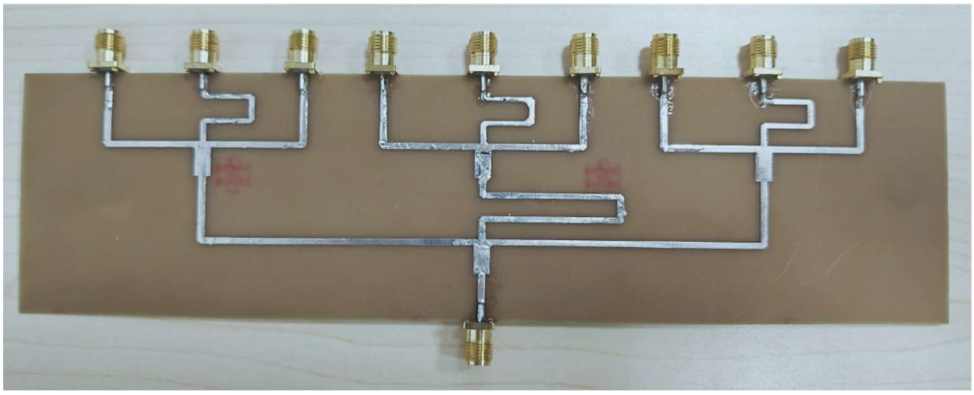

Fig. S13. 1-to-9 power divider performance

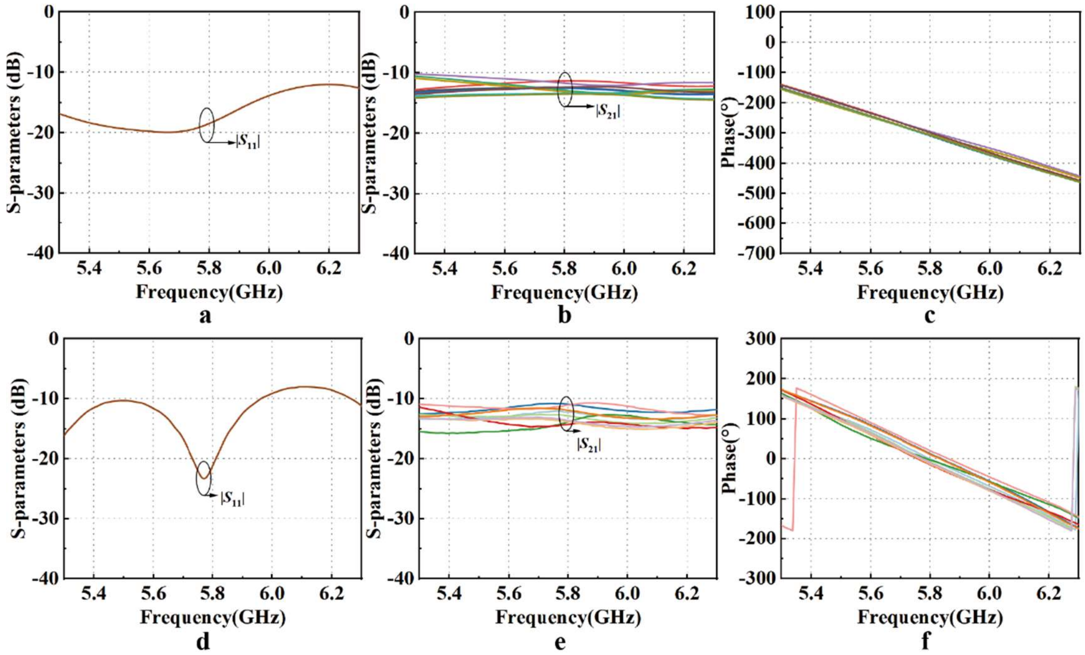

Fig. S14 1-to-9 power divider performance. Simulated results: (a) reflection coefficients, (b) transmission coefficients, and (c) transmission phase. Measured results: (d) reflection coefficients, (e) transmission coefficients, and (f) transmission phase.

Table S6

Case 1: simulated results of power divider at 5.8 GHz

Port	1	2	3	4	5	6	7	8	9
Mag.	0.78	1.0	0.76	0.70	0.91	0.65	0.62	0.78	0.60
Phase(°)	0	10.76	1.49	4.13	14.55	3.58	1.36	10.96	2.97

Table S7

Case 2: measured results of power divider at 5.8 GHz

Port	1	2	3	4	5	6	7	8	9
Mag.	0.74	1.0	0.64	0.51	0.97	0.46	0.51	0.80	0.54
Phase(°)	0	12.35	7.70	3	19.91	14.90	15.70	10.24	9.90

Table S8

Case 3: port phase is consistent and the amplitude is different at 5.8 GHz

Port	1	2	3	4	5	6	7	8	9
Mag.	0.12	0.63	0.15	0.67	0.93	0.78	0.76	0.39	0.71
Phase(°)	0	0	0	0	0	0	0	0	0

Table S9

Case 4: port phase is different and the amplitude is the same at 5.8 GHz

Port	1	2	3	4	5	6	7	8	9
Mag.	1	1	1	1	1	1	1	1	1
Phase(°)	47	70	34	57	3	32	52	74	77

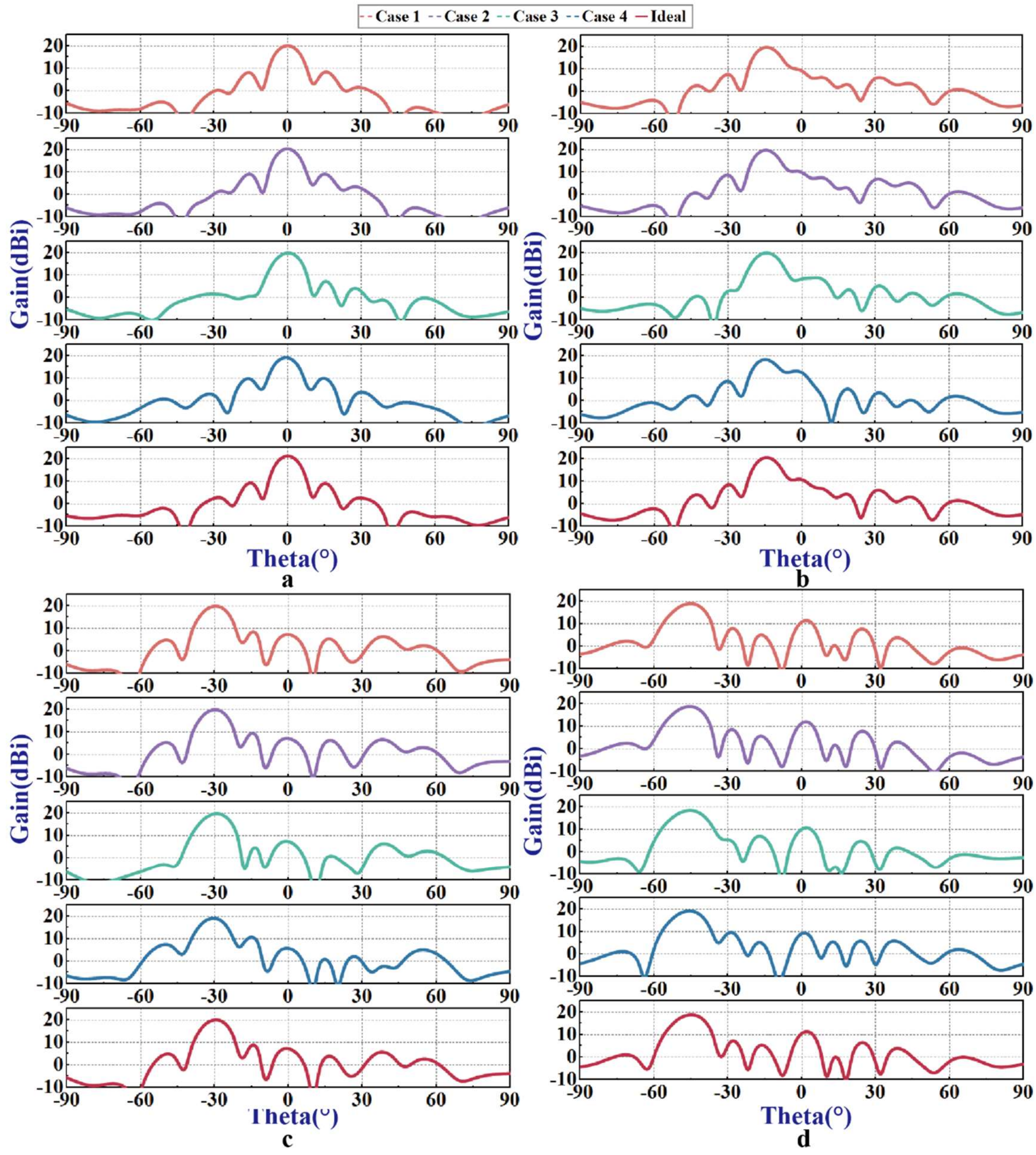

Fig. S15 Beam scanning characteristics of RIS in radiation mode when nine ports are fed with different RF signals. (a) 0°. (b) -15°. (c) -30°. (d) -45°.

Section S12: Wireless Energy Harvesting

The proposed RIS can achieve wireless energy harvesting. The metasurface array has a total of nine RF ports. When the PIN diodes are in the 00 state, the proposed RIS can perform wireless energy harvesting. The reflection coefficients of these nine RF ports are shown in Fig. S16. It can be seen that the reflection coefficients of these nine ports are below -10 dB in the frequency range

of 5.6-5.95 GHz, indicating a matched state. Therefore, it can serve as an effective antenna for wireless energy harvesting.

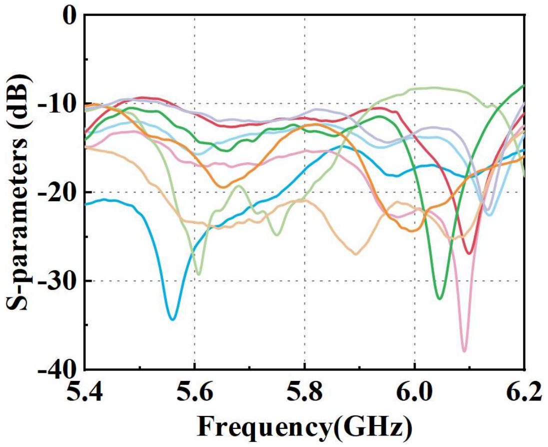

Fig. S16. Reflection coefficient in wireless energy harvesting mode.

Section S13: Beam Scanning in Scattering Mode

In the scattering mode, the beam-scanning results at other frequencies obtained through simulation are shown in Fig. S17. It can be observed that single-beam scanning of ±45° is achievable at both 5.75 GHz and 5.85 GHz. Single-beam scanning of ±45° is also achievable in both the E-plane and H-plane. While the side-lobe level is slightly lower for large-angle beam scanning, the side-lobe level for other angles is greater than 10 dB. Therefore, the introduction of initial phases in the 1-bit scattering RIS can suppress grating lobes during beam scanning. Moreover, the quantized initial phases also effectively enhance the side-lobe level during beam scanning.

In the scattering mode, the beam-scanning results at other frequencies obtained through testing are shown in Fig. S18. It can be seen that single-beam scanning of ±45° is achievable at both 5.75 GHz and 5.85 GHz. These results are consistent with the simulation outcomes. The side-lobe level is slightly lower for large-angle beam scanning, while for other angles, the side-lobe level is greater than 10 dB.

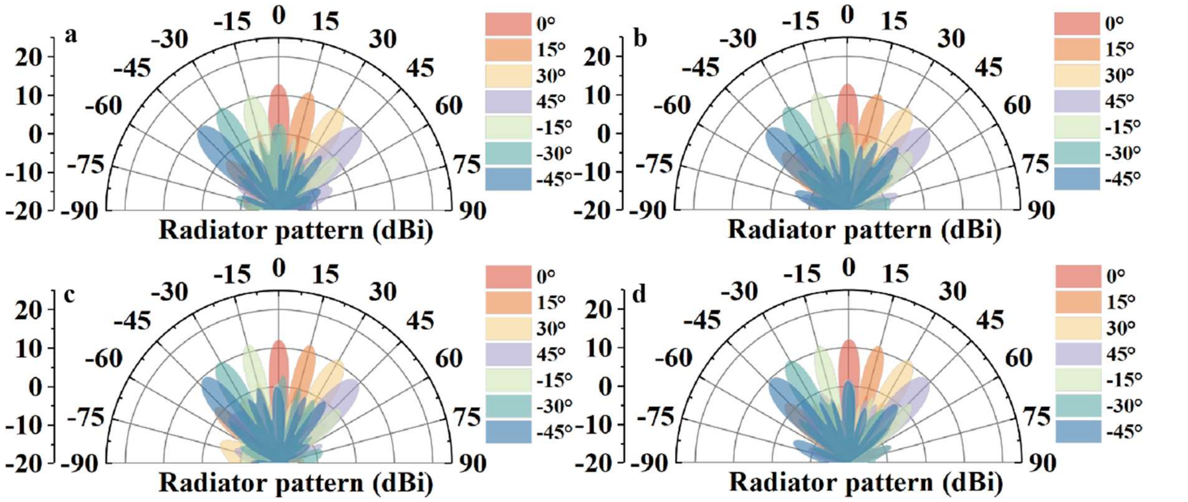

Fig. S17. Simulated beam scanning results. (a) 5.75 GHz for E-plane. (b) 5.75 GHz for H-plane. (c) 5.85 GHz for E-plane. (d) 5.85 GHz for H-plane.

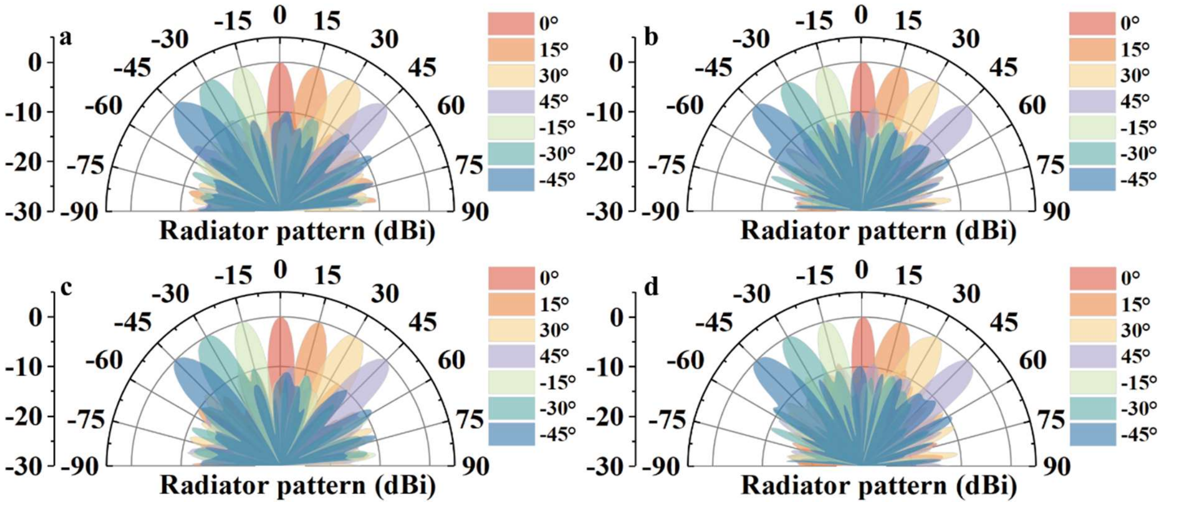

Fig. S18. Measured beam scanning results. (a) 5.75 GHz for E-plane. (b) 5.75 GHz for H-plane. (c) 5.85 GHz for E-plane. (d) 5.85 GHz for H-plane.

Section S14: Beam Scanning without Initial Phase in Scattering Mode

To highlight the advantages of initial phase design, we present the beam characteristics of RIS without initial phase. Figure S19 illustrate the beam-scanning characteristics of RIS in scattering mode, respectively. Figures S19a and b show the E-plane beam-scanning characteristics at positive angles in scattering mode without and with initial phases, respectively. It can be observed that the absence of initial phases results in dual-beam scanning. Moreover, the beam-scanning sidelobes from 0° to ±45° are significantly large, with the sidelobes at ±45° almost matching the main lobe. Specifically, the gains at 0°, ±15°, ±30°, and ±45° are 14.01 dBi, 11.31 dBi, 11.02 dBi, and 8.43

dBi, respectively. It is evident that the gain of the scanned beam drops rapidly. In contrast, with the initial phase, RIS achieves single-beam scanning. The sidelobes are maintained below 1.5 dB. The gains at 0°, 15°, 30°, and 45° are 13.33 dBi, 12.01 dBi, 11.32 dBi, and 10.31 dBi, respectively. Although the array's broadside gain is reduced with the initial phase, the scanned beam gain and sidelobe levels are superior to those without the initial phase. Figures S19c and d depict the H-plane beam-scanning characteristics at positive angles without and with initial phases, respectively. It can be observed that the H-plane exhibits similar characteristics. Therefore, the distinction between using and not using initial phases goes beyond merely suppressing grating lobes. With initial phases, the scanning gain of the array is enhanced, and sidelobe levels are reduced.

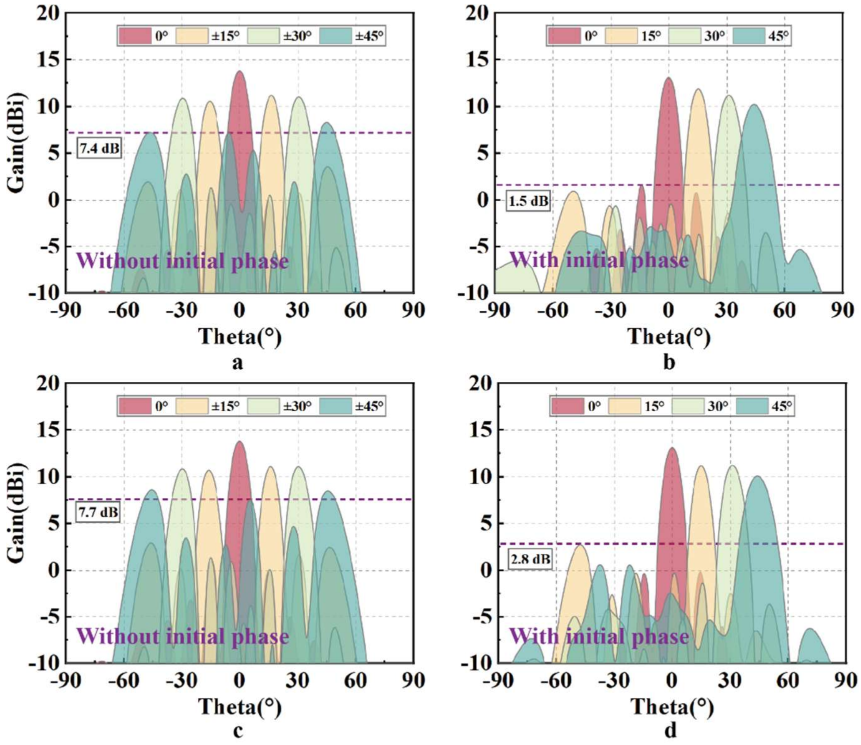

Fig. S19 Beam scanning in scattering mode. E-plane scanning (a) with, and (b) without initial phase. H-plane scanning (c) with, and (d) without initial phase.

Section S15: Materials and Methods

Numerical simulation: The S-parameter performance of meta-atom, and far-field radiation and scattering properties of the RIS are computed through full-wave numerical simulations with the finite element method solver in high-frequency simulation software (HFSS) of Ansys Electronics Desktop 2022. During the numerical simulation, the PIN diode and capacitors are simulated with

an equivalent circuit. In the ON state, the equivalent circuit of the PIN diode is a series combination of a 0.8 Ω resistor and a 0.45 nH inductor. In the OFF state, it is equivalent to a series combination of a 0.21 pF capacitor and a 0.45 nH inductor. NLOS wireless communication scenario is simulated by WinProp software. The WinProp software employs ray-tracing algorithms to predict the propagation characteristics of electromagnetic waves. It is primarily composed of modules such as WallMan, AMan, and ProMan. The WallMan module is used to model the L-shaped scenario of the school's building. The AMan module is used for format conversion and import of the RIS radiation pattern. Finally, the ProMan module is used to perform wave propagation and blind-spot coverage power analysis for the L-shaped scenario. Since the ProMan module only supports the setup of a single antenna as the radiation source, it cannot handle the radiation source mode combining the base station and RIS. Therefore, the response of the RIS to the base station is calculated through the communication link, and the EIRP is set accordingly. This simulates the combined effect of the two radiation sources.

Experimental measurement: We conducted three experiments in the microwave chamber, indoor and L-shaped corridor environments to evaluate the RIS's performance. The measurement in the microwave chamber mainly contains a receiving standard horn antenna LB-20180 from INFO company and a vector network analyzer N5244A from KEYSIGH company for far-field beam evaluation of RIS. In the far-field measurement, the receiving horn records the outgoing wave intensity from the RIS. The RIS is fixed on a rotation table to record the radiation and scattering electrical field with different angles. We recorded the output power from a standard horn antenna as a reference to calculate the pattern. In the indoor experiment, two measurement devices are set up in the laboratory to verify the wireless communication and WEH. The measurement in wireless communication contains a standard horn antenna LB-20180, a 1-to-9 divider, two software-defined radios, and two laptops. One USRP was used to connect to the input port of the RIS, while the other USRP was connected to the receiving horn antenna. The measurement in the WEH contains a standard horn antenna LB-20180, a signal generator, a power meter, and a laptop. The signal generator generates a 5.8 GHz signal that is received by the RIS. The power values received by the 9 RF ports of the RIS are then read through the power meter. In an L-shaped corridor experiment, NLOS communication measurement contains a vector network analyzer (Agilent N9918A), a signal generator, a transmitter horn, and a receiving horn. The transmitter horn transmits the signal to the RIS. The receiver horn is reached after RIS scattering regulation. The

received power value is also recorded through the spectrometer mode of the vector network analyzer.

Section S16: Performance Comparison

The proposed radiation-scattering RIS application scenarios can be divided into three types. They are wireless communication, NLOS wireless communication, and communication-sensing integration. Communication-sensing integration refers to the integration of LOS and NLOS communication. Tables S10-S12 provide a performance and cost analysis of the proposed RIS compared to existing RIS technologies for the three application scenarios. From the above three application scenarios, it can be inferred that the proposed RIS has low-cost characteristics. Although other RIS also have advantages in LOS wireless communication and NLOS wireless communication. However, in communication-sensing integration, the proposed RIS is the best low-cost design in all aspects.

For wireless communication scenarios, Table S10 shows the cost and performance comparison with existing studies. Table S10 covers the major categories of RIS commonly used in wireless communication. These are reflection RIS and radiation RIS. The comparison is made in terms of profile, number of PIN diodes, number of I/O ports, energy, performance, and aperture efficiency. Among these, the number of PIN diodes, the number of I/O ports, and energy focus on individual elements. The energy loss of the entire array can be partially understood from this. In addition, the commonly used PIN diode models are SMP 1340 and MADP 000907. The minimum conduction energy for these PIN diodes is 0.38 mW and 0.5 mW, respectively. These two types of PIN diodes can be used universally in low-frequency bands. Therefore, disregarding the type of PIN diode, energy loss primarily depends on the number of PIN diodes. It can be seen that the proposed RIS is more efficient than 1-bit reflection and 1-bit radiation RIS (with initial phase). The proposed RIS is comparable in efficiency to 2-bit radiation RIS but slightly less efficient than 2-bit reflection RIS. Thus, in terms of efficiency, the proposed RIS is comparable to 2-bit radiation RIS. Therefore, introducing an initial phase not only suppresses grating lobes but also improves efficiency and sidelobe levels. However, compared to 2-bit radiation RIS, the proposed RIS has a lower cost. Although the 2-bit reflection RIS has the best efficiency, its profile height reaches 13.47λ. Therefore, it is not the best choice. Higher-bit RIS, such as 3-bit and 4-bit, have smaller quantization errors and higher efficiency. However, the introduction of a large number of switches

leads to switch losses that cannot be ignored. Thus, the 1-bit RA is the lowest-cost option when profile and efficiency are not considered. It requires the smallest number of PIN diodes, I/O ports, and energy. Considering profile and efficiency, the proposed RIS is the best choice.

Table S10

Wireless communication scenario

Type	Profile	PIN diodes	I/O	Power	Performance	Efficiency
This work	0.04λ	2 (SMP1340)	2	0.76 mW	Single beam scanning/ Low cost	24.7% (High)
1-bit RA[10]	24λ	1 (MADP000907)	1	0.5 mW	Single beam scanning/ Minimum cost	12.15% (Low)
2-bit RA[11]	13.47λ	2 (MADP000907)	2	1.0 mW	Single beam scanning/ Low cost	27.1% （High）
1-bit PPA[43]	0.08λ	2 (MADP000907)	2	1.0 mW	Single beam scanning/ Low cost (with initial phase)	22.9% (High)
2-bit PPA[38]	0.11λ	4 (MADP000907)	4	2.0 mW	Single beam scanning/ High cost	24.3% (High)

Reflectarray: RA Programmable phased array: PPA

For NLOS wireless communication scenarios, Table S11 shows the cost and performance comparison with existing studies. In this scenario, RIS acts as a relay, forwarding base station signals to coverage holes. Table S11 covers the RIS used in NLOS wireless communication. Since NLOS wireless communication requires scattered signals, only reflection RIS can be used. Table S2 makes the comparison in terms of profile, number of PIN diodes, number of I/O ports, energy, and performance. In NLOS wireless communication scenarios, due to plane wave incidence, the aperture efficiency is very low. Therefore, its efficiency is not measured. However, as shown in Figs. S12 and S19, the gain of single-beam scanning is larger than that of dual-beam scanning. Thus, the efficiency of single-beam is higher. Thus, the efficiency relationship in Table S11 is: Proposed RIS≈2-bit RA>1-bit RA. Among these, the number of PIN diodes, the number of I/O ports, and energy focus on individual elements. The energy loss of the entire array can be partially understood from this. It can be seen that the proposed RIS and 2-bit RA are the best choices.

Table S11

NLOS wireless communication scenario

Type	profile	PIN diodes	I/O	Power	Performance
This work	0.04λ	2 (SMP1340)	2	0.76 mW	Single beam scanning/ High efficiency/Low cost
1-bit RA[10]	0.08λ (PW)	1 (MADP000907)	1	0.5 mW	Dual beam scanning/Low efficiency/ Minimum cost
2-bit RA[11]	0.14λ (PW)	2 (MADP000907)	2	1.0 mW	Single beam scanning/ High efficiency/ Low cost

Reflectarray: RA Programmable phased array: PPA

For the integrated communication and sensing scenario, Table S12 shows the cost and performance comparison with existing studies. This scenario integrates the aforementioned wireless communication and NLOS wireless communication. In this scenario, RIS can transmit signals autonomously or relay signals from other base stations. Table S12 presents several designs for integrated communication and sensing. To achieve integrated communication and sensing, existing technologies can use a patchwork approach. For example, integrating 1-bit RA with 1-bit PAA. Or integrating 2-bit RA with 2-bit PAA. Table S12 compares the designs in terms of the number of PIN diodes, number of I/O ports, hardware resources, and performance. Hardware resources refer to the number of PIN diodes, number of I/O ports, energy, PCB cost, hardware system, etc. Due to inconsistencies in the number of PIN diodes and I/O ports and energy loss across the designs. They are compared separately. Excluding the above factors, hardware resources involve several relatively uniform aspects, such as PCB area. Thus, they are combined into a single entry. And the multiples increased compared to the proposed RIS are recorded. As can be seen, the proposed RIS achieves the lowest cost with the fewest PIN diodes, I/O ports, energy loss, and hardware resources when considering profile and efficiency. Thus, from the above three application scenarios, it can be concluded that the proposed RIS has a low cost. Although other RIS also have advantages in wireless communication and NLOS wireless communication. However, in integrated communication and sensing, the proposed RIS is the best low-cost design in all aspects.

Table S12

Integrated communication and sensing scenario

Type	PIN	I/O	Power	Hardware	Performance
This work	2	2	0.76 mW	1	Single beam scanning/ high efficiency/ Low cost
1-bit RA[10]/ 1-bit PAA[43]	3	3	1.14 mW	2	Dual beam scanning/ Low efficiency/ Low cost
2-bit RA[11]/ 2-bit PAA[38]	4	4	1.52 mW	2	Single beam scanning/ high efficiency/ High cost
RSRA[45]	3	3	1.14 mW	1	RA: Dual beam scanning/ Low efficiency PA: Single beam scanning/ high efficiency/High cost
RSRA[47]	4	4	1.52 mW	1	RA: Dual beam scanning/ Low efficiency PA: Single beam scanning/ high efficiency/High cost

Reflectarray: RA Programmable phased array: PPA Radiation-scattering reconfigurable array: RSRA.
